# Associations between the EAT-Lancet planetary health diet and incident dementia

**DOI:** 10.1016/j.tjpad.2025.100166

**Published:** 2025-04-12

**Authors:** Jessica Samuelsson, Isabelle Glans, Anna Stubbendorff, Ulrika Ericson, Sebastian Palmqvist, Oskar Hansson, Emily Sonestedt

**Affiliations:** a Neuropsychiatric Epidemiology Unit, Department of Psychiatry and Neurochemistry, Institute of Neuroscience and Physiology, Sahlgrenska Academy, Centre for Ageing and Health at the University of Gothenburg, Wallinsgatan 6, Mölndal 43139, Sweden; b Clinical Memory Research Unit, Department of Clinical Sciences Malmö, Lund University, S:t Johannesgatan 8, Malmö, Sweden 21146; c Memory Clinic, Skåne University Hospital, S:t Johannesgatan 8, 21146, Malmö, Sweden; d Nutritional Epidemiology, Department of Clinical Sciences Malmö, Lund University, Jan Waldenströms gata 35, Malmö 21428, Sweden; e Diabetes and Cardiovascular Disease – Genetic Epidemiology, Department of Clinical Science Malmö, Jan Waldenströms gata 35, Malmö 21428, Sweden; f Department of Food and Meal Science, Faculty of Natural Science, Kristianstad University, Kristianstad 21939, Sweden

**Keywords:** Planetary health diet, EAT-Lancet diet, Dementia, Alzheimer's disease, Vascular dementia

## Abstract

**Background:**

The impact of the environmentally sustainable EAT-Lancet diet on dementia risk remains poorly understood. The aim was to investigate associations between the EAT-Lancet diet and incident dementia.

**Methods:**

Associations of the EAT-Lancet diet with all-cause dementia, Alzheimer's disease (AD), and vascular dementia (VaD) were investigated among 25,898 participants from the Malmö Diet and Cancer study, Sweden. Participants aged 45–73 years were recruited for the baseline examination between 1991 and 1996, and the mean follow-up time was 18 years. To assess robustness of estimations, we used seven previously constructed EAT-Lancet diet scores. Multi-adjusted Cox proportional hazard analyses were performed, with results presented per 10 % in increment scores. Additionally, we explored the potentially modifying effect of *APOE* ε4 status in this context.

**Results:**

With one of the scores, higher adherence to the EAT-Lancet diet was associated with a reduced risk of AD and all-cause dementia. Moreover, the results suggest an interplay between the EAT-Lancet diet and *APOE* ε4 status. A risk-reducing effect was observed among *APOE* ε4 non-carriers with three of the scores in relation to AD, and with five of the scores in relation to all-cause dementia. No associations were observed among *APOE* ε4 carriers, or in relation to VaD.

**Conclusion:**

The results indicate a risk reducing effect of adhering to the EAT-Lancet diet among *APOE* ε4 non-carriers, and no negative effects on dementia risk were detected. Future studies should consider the potentially modifying effect of *APOE* ε4 status, and the implications of methodological differences in measuring adherence to the EAT-Lancet diet.

## Highlights

• EAT-Lancet diet adherence was not associated with increased dementia risk.
• *APOE* ε4 modified the impact of the EAT-Lancet diet on Alzheimer's disease (AD) risk.
• The EAT-Lancet diet reduced the risk of AD and dementia among *APOE* ε4 non-carriers.

## 1 Introduction

With global populations ageing, dementia cases are estimated to increase worldwide [1]. The Lancet commission on dementia prevention has listed 14 potentially modifiable risk factors that account for about 45 % of worldwide dementias, including lifestyle factors such as physical inactivity, excessive alcohol consumption, smoking, and obesity [2]. While diet was not included as a risk factor in the report, it has been associated with dementia risk [2,3]. Healthy dietary patterns such as the Mediterranean diet (MeDi) have been shown to slow down cognitive decline and reduce dementia risk [4]. However, results from previous studies are inconsistent [2,5].

In 2019, the EAT-Lancet commission presented a healthy reference diet aimed to be environmentally sustainable [6]. The diet is constructed to keep the environmental impact within planetary boundaries, provide adequate amounts of nutrients, reduce global malnutrition, and reduce the incidence of non-communicable diseases such as obesity, diabetes and cardiovascular disease [6]. The EAT-Lancet diet is mainly plant-based with a limited intake of animal-based foods such as fish, dairy and meat, and is constructed to be adaptable to globally diverse culinary traditions [6]. Research has shown that higher adherence to the diet may reduce the risk of mortality [7], stroke [8], coronary events [9], and diabetes type 2 [[10], [11], [12]]. However, its impact on dementia risk remains largely unexplored [13]. Furthermore, it has been suggested that higher adherence to the diet may have negative consequences on brain health by inflicting nutrient deficiencies [14]. With an increased focus on incorporating environmental sustainability into general dietary guidelines, it is crucial to explore the impact of this diet on dementia risk.

Methodological discrepancies in scoring methods to assess adherence to the MeDi may partly account for inconsistencies in previous studies [15]. Similar challenges may also influence research on other diets, such as the EAT-Lancet diet [16]. A variety of EAT-Lancet diet scores have been constructed, and seven of these scores have been compared and estimated in relation to all-cause mortality, stroke, and greenhouse gas emissions [16]. The authors concluded that the scores had differences in construction, interpretation, and relation to disease and climate-related outcomes, and recommended the use of several scores to assess robustness of estimations when exploring links between the EAT-Lancet diet and health outcomes [16].

To incorporate environmental sustainability into dietary guidelines in dementia prevention strategies, it is essential to understand how planetary health diets, such as the EAT-Lancet diet, influence dementia risk. While Mediterranean-style dietary patterns have been extensively studied in this context, the effects of the EAT-Lancet diet on dementia risk remains poorly understood. Our hypothesis was that the environmentally sustainable EAT-Lancet diet does not have negative effects on brain health. We investigated associations between the EAT-Lancet diet and incident Alzheimer's disease (AD), vascular dementia (VaD), and all-cause dementia in a Swedish cohort, with a mean follow-up time of 18 years. Furthermore, we investigated the potentially modifying effect of *APOE* ε4 status in this context. Additionally, we investigated associations between the diet and amyloid-β 42 (Aβ42) pathology. To investigate robustness of estimations, adherence to the EAT-Lancet diet was measured with seven previously constructed dietary scores, with results presented per 10 % in increment scores.

## 2 Methods

### 2.1 Study sample

**Fig. 1:**
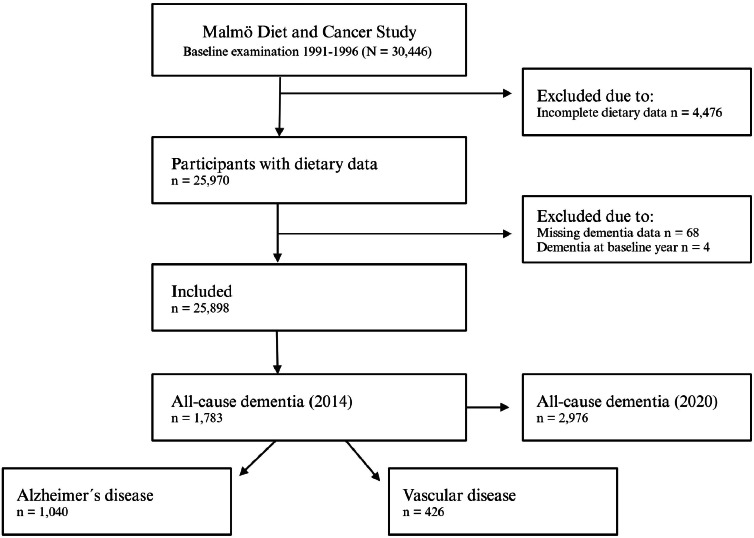
Participant flowchart of the Malmö Diet and Cancer study.

Data was derived from the Swedish Malmö Diet and Cancer Study (MDCS). Participants aged 45–73 years were recruited for the baseline examination between 1991 and 1996. The exclusion criteria were inability to speak Swedish and intellectual disability (eligible sample: *n* = 68,905). The recruitment process of the MDCS has been described previously [17,18]. In total, 30,446 individuals participated. Participants with incomplete dementia or dietary data were excluded. The final sample included 25,898 participants. The participant flowchart can be found in Fig. 1.

### 2.2 Dietary assessment

Information on dietary intake was assessed using a validated, modified diet history method consisting of a 7-day food diary, a 168-item food frequency questionnaire (FFQ) covering consumption frequencies and portion sizes of habitual food intake during the preceding 12 months, and a 45–60-minute dietary interview with trained personnel, described previously [7,19]. The average total food intake (g/day) was summarised from the food diary and the FFQ. Extreme values of total energy, nutrients, major food groups, and portion sizes were checked for errors. Energy and nutrient intakes were calculated using a food composition database (PC-KOST2–93) from the Swedish National Food Agency (1600 food items).

### 2.3 EAT-Lancet reference diet scores

Adherence to the EAT-Lancet diet was calculated with seven previously constructed dietary scores [7,12,[20], [21], [22], [23], [24]]. Six of the scores were identified in a previous study, and the seventh score was constructed based on a study by Bui et al. [16,24]. The reason for including seven different scores was to explore the robustness of associations between the EAT-Lancet diet and incident dementia by considering different interpretations and scoring systems of the EAT-Lancet diet. The scores included individual components highlighted in the EAT-Lancet reference diet but differed in construction, scoring system, and possible score range (supplementary figure 1, and supplementary Table 1). The scores were labelled based on the first author of the articles describing the scores. Colizzi et al. [23], Trijsburg et al. [20], Kesse-Guyot et.al [22], and Bui et al. [24] used a proportional scoring system, Knuppel et al. [12], and Hanley-Cook et al. [21] a binary system, and Stubbendorff et al. [7] an ordinal scale system. All scores were transformed for comparability, with results presented per 10 % in increment score (range; 0 (nonadherence) to 10 (high adherence)).

### 2.4 Dementia

All-cause dementia, and subtypes of dementia were based on registered dementia diagnoses from the Swedish National Patient Register (NPR) throughout 2020. The NPR covers both the Swedish Inpatient Register and the hospital-based outpatient register. The dementia diagnoses have been validated by trained physicians based on symptoms, results of cognitive tests, and brain imaging in accordance with the DSM-5 (Diagnostic and Statistical Manual of Mental Disorders, Fifth Edition) criteria throughout 2014, described previously [19]. In cases where cerebrospinal fluid (CSF) concentrations of Aβ42 and tau phosphorylated at Thr181 were available, AD diagnosis was based on the NIA-AA criteria [25]. There were three binary outcome variables for dementia (yes/no): all-cause dementia, VaD, and AD (pure and mixed with vascular pathology). In sensitivity analyses, we included all-cause dementia throughout 2020 with validated diagnoses throughout 2014, and unvalidated diagnoses between 2015 and 2020. Unvalidated diagnoses were based on the following registered diagnostic codes: AD dementia (ICD-9 and ICD-10 codes F00, G30, 331A/331.0, VaD (F01, 290E/290.4), Parkinson's disease dementia (F020, G310, 331B/331.1), or unspecified dementia (F03, 290, 294B/294.1, 331C/331.2) [26].

### 2.5 Genetic risk

The apolipoprotein E (APOE) gene is the strongest genetic factor associated with the risk of developing sporadic AD [27]. There are three common alleles of the *APOE* gene: the protective *APOE* ε2 allele, the neutral *APOE* ε3 allele, and the risk increasing *APOE* ε4 allele. We included data on *APOE* ε4 status (carrier/non-carrier). Out of the included study sample, there were 24,987 individuals with data on *APOE* ε4 status (17,444 non-carriers, 7543 carriers).

### 2.6 Amyloid-β 42

Accumulation of amyloid-β (Aβ) is related to AD, and can be detected with cerebrospinal fluid (CSF) analysis of Aβ42 or Aβ PET imaging [28,29]. CSF data were available for participants with signs of cognitive decline who had been referred to the Memory Clinic at Skåne University Hospital for further investigation. CSF was collected between 1995 and 2015. A total of 777 (669 with dietary data) participants with signs of cognitive decline underwent lumbar puncture [19]. Concentrations of cerebrospinal fluid (CSF) Aβ42 were measured using INNOTEST ELISA (Fujirebio Europe, Ghent, Belgium). Due to a slight, assay-dependent drift in levels of CSF Aβ42 during the collection period 1995–2015, two different cutoffs for pathology were established for the period 1995–2003 (Aβ42 < 484.8 pg/mL) and 2004–2015 (Aβ42 < 577.1 pg/mL) [26].

### 2.7 Characteristics and potential confounders

Characteristics and potential confounders were collected at baseline examination with a self-administrated questionnaire, and with anthropometric measurements. Body mass index (BMI) was calculated as kg/m^2^. Leisure-time physical activity was assessed as time spent per week on 17 different activities, with results multiplied by their respective intensity factor. This was added to a total and the participants were divided into quintiles of leisure-time physical activity. Alcohol consumption was dichotomised with a cutoff for excessive alcohol consumption at >24 *g*/day [2]. Smoking was divided into current smoking, previous smoking, or never smoking. Education level was divided into primary education (≤8 years), secondary education (9–12 years), or higher education/university (≥13 years). Season was divided into winter, spring, summer, and fall. A binary variable labelled “diet method version” divided participants with either a 60-minute or a 45-minute dietary interview. Diabetes included prevalent and incident diabetes (until 2014).

### 2.8 Statistics

Continuous variables were presented as means, and proportions as number and percent (%). Comparisons of characteristics between participants with and without dementia were investigated with either the student's independent t-test, Mann-Whitney U test, or Chi-square test. Pearson correlation coefficient analyses were performed between the EAT-Lancet diet scores.

Cox proportional hazard models, with years between baseline and event as time variable, were used to examine associations between the EAT-Lancet diet scores and incident dementia. The Cox proportional hazard assumption was tested based on Schoenfeld residuals. Event was defined as all-cause dementia, VaD, or AD. Censoring occurred at the recorded date of dementia, time of death, or at time of register data delivery (December 31, 2014). Furthermore, sensitivity analyses were performed with all-cause dementia with time of register data delivery December 31, 2020. The analyses were performed in two models. Model 1 was adjusted for age, sex, season, dietary sampling method, and energy intake. Model 2 was adjusted for age, sex, season, dietary sampling method, energy intake, education, smoking, alcohol consumption, physical activity, and BMI. We added education as a middle step in between model 1 and 2 to explore the impact of education on the associations.

In addition, we compared participants with the highest adherence (Q5) to participants with the lowest adherence (Q1) in relation to all-cause dementia (2014), AD, and VaD, in models adjusted for potential confounders (model 2). The quintile categorization was performed based on tied values. This was done as dividing the participants into even numbered quintiles would produce categories were participants with the same value of adherence could be divided into different categories (for some of the scores).

Gene-diet interactions were explored with Cox proportional hazard analyses for all seven dietary scores by entering the interaction variables (EAT-Lancet diet score* *APOE ε4* status) as predictors and AD, VaD, and all-cause dementia (2014) as outcome variables, adjusted for potential confounders (model 1 and 2).

#### 2.8.1 Additional analyses

Associations between the EAT-Lancet diet (seven scores) and Aβ42 pathology (yes/no) were investigated with binary logistic regression models, adjusted for potential confounders (model 1 and 2).

We conducted a non-parametric bootstrap analysis (1000 resamples) to compare the effects of seven dietary scores on incident dementia using Cox proportional hazards models adjusted for potential confounders (model 2). For each bootstrap iteration, log hazard ratios (log HRs) were estimated for all scores. Pairwise differences in log HRs were computed, and significance was assessed using two-sided bootstrap p-values.

#### 2.8.2 Sensitivity analyses

We performed sensitivity analyses excluding BMI as a confounder (potential mediator). Additionally, we performed analyses were those with diabetes were excluded (potential changes in dietary intake). Furthermore, we performed analyses were those with incident dementia within five years from baseline examination were excluded (potential preclinical/prodromal stage).

The statistical analyses were performed in IBM SPSS STATISTICS 29, and R programming version 4.1.2. For the interaction analyses, a *p*-value threshold of <0.1was set to detect an interaction [30]. For all other analyses, a *p*-value <0.05 was considered significant.

## 3 Results

### 3.1 Characteristics

There were 25,898 participants with dietary and dementia data. Out of these, 1,783 (6.9 %) developed dementia by 2014 (*n* = 1,040 with AD, and 426 with VaD), and 2,976 (11.5 %) developed dementia by 2020. Mean follow up-time until 2014 was 18.0 years (SD 4.9 years, 46,7168 person-years). Mean age at baseline was higher amongst participants that developed dementia compared to those that did not (dementia, 2014: 64.5 vs. 57.7 years), (dementia, 2020: 62.8 vs. 57.6 years). Baseline characteristics can be found in Table 1.

**Table 1:** Baseline characteristics of the Malmö diet and cancer study population.

	Total *N* = 25,898	Dementia (2014) *N* = 1,783	No dementia (2014) *N* = 24,115	*P*-value
	Mean (SD)	Mean (SD)	Mean (SD)	
EAT-Lancet diet scores^a^				
Knuppel	6.33 (0.96)	6.28 (0.94)	6.34 (0.96)	0.0092
Trijsburg	4.03 (1.09)	4.04 (1.09)	4.03 (1.09)	0.34
Hanley-Cook	3.00 (1.06)	3.00 (1.04)	3.00 (1.06)	0.97
Kesse-Guyot	3.55 (0.82)	3.53 (0.78)	3.55 (0.83)	0.28
Stubbendorff	4.32 (0.81)	4.36 (0.78)	4.32 (0.81)	0.048
Colizzi	3.68 (0.92)	3.71 (0.91)	3.68 (0.92)	0.089
Bui	5.59 (0.73)	5.62 (0.69)	5.59 (0.73)	0.021
Baseline age, years	58.2 (7.7)	64.5 (6.0)	57.7 (7.6)	<0.0001
BMI^b^	25.8 (4.0)	26.1 (4.0)	25.7 (4.0)	<0.0001
Energy intake, kJ/day	9,481 (2,706)	9,323 (2,738)	9,492 (2,703)	0.011
Follow-up time^c^	18.0 (4.9)	14.4 (4.7)	18.3 (4.8)	NA
	**N ( %)**	**N ( %)**	**N ( %)**	***P*-value**
Sex assigned at birth, female	15,775 (61)	1098 (62)	14,677 (61)	0.55
*APOE ε4* carriers	7,543 (30)	881 (51)	6,662 (29)	<0.0001
Diabetes^d^	4,823 (19)	371 (21)	4,452 (19)	0.015
Education				<0.0001
<8 years	10,800 (42)	940 (53)	9,860 (41)	
9–12 years	9,099 (35)	590 (33)	8,509 (35)	
≥13 years	5,999 (23)	253 (14)	5,746 (24)	
Physical activity^b^				0.065
1	5,098 (20)	333 (19)	4,765 (20)	
2	5,103 (20)	355 (20)	4,748 (20)	
3	5,155 (20)	324 (18)	4,831 (20)	
4	5,154 (20)	370 (21)	4,784 (20)	
5	5,268 (20)	392 (22)	4,876 (20)	
Alcohol, <24 *g*/day	22,889 (88)	1,624 (91)	21,265 (88)	0.0002
Smoking^b^				<0.0001
Current smoker	7,360 (28)	375 (21)	6,985 (29)	
Previous smoker	8,809 (34)	619 (35)	8,190 (34)	
Never smoker	9,721 (38)	789 (44)	8,932 (37)	

*Note:* Comparisons of characteristics between participants with and without dementia were investigated with the student's independent t-test for continuous variables (dietary scores, age, BMI, and kJ/day), Mann-Whitney U test (education and physical activity) or Chi-square test for categorical variables (alcohol, *APOE* ε4 status, smoking, diabetes, and biological sex).

a The scores were labelled based on first authors of the articles reporting the construction of the scores. Adherence was reported per 10 % in increment score for comparability.

b There were missing data on smoking (*n* = 8), BMI (*n* = 40), and physical activity (*n* = 120). In total 0.64 % (*n* = 165) of cases.

c Time from baseline to dementia, or until death or end of study for those that did not develop dementia (2014).

d Prevalent or incident diabetes during study period (2014).

### 3.2 The EAT-Lancet diet scores

Out of the seven scores, the Hanley-Cook score provided the lowest mean score (3.00), and the Knuppel score the highest (6.33) (supplementary fig. 2, Table 1). The score averages were similar amongst participants with and without dementia (Table 1). A correlation matrix of the dietary scores can be found in supplementary figure 3. The lowest correlation was found between the Kesse-Guyot and the Hanley-Cook scores (*r*: 0.26), and the highest between the Bui and the Stubbendorff scores (*r*: 0.83).

### 3.3 Associations between the EAT-Lancet diet and all-cause dementia

**Fig. 2:**
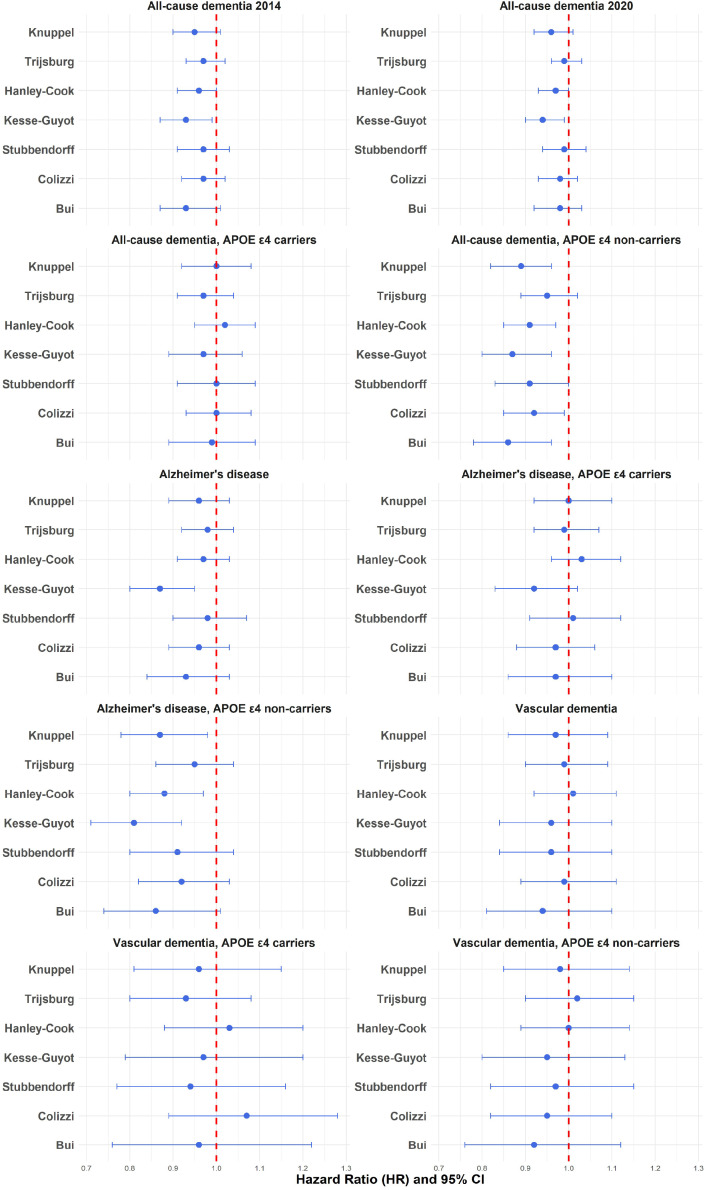
The EAT-Lancet diet in relation to incident dementia. The figure reports associations between the EAT-Lancet diet measured with seven EAT-Lancet diet scores in relation to all-cause dementia, Alzheimer's disease and vascular dementia for the total population, and by *APOE* ε4 status (carrier/non-carrier). Associations were investigated with Cox proportional hazard models adjusted for age, sex, season, diet method version, energy intake, education, smoking, alcohol consumption, physical activity, and BMI. Results are presented per 10 % in increment scores. The EAT-Lancet scores were labelled based on first authors of the articles that reported the construction of the scores. All-cause dementia was followed from baseline until 2014 (validated diagnoses) and 2020 (validated diagnoses until 2014, and unvalidated diagnoses between 2015 and 2020). Alzheimer's disease (AD) and vascular dementia (VaD) were followed from baseline until 2014. Evidence of interactions between the EAT-Lancet diet and *APOE* ε4 status was detected in relation to all-cause dementia and AD, but not in relation to VaD, reported in supplementary Tables 5–7. The EAT-Lancet diet*APOE ε4 interaction analyses were performed in relation to the validated dementia diagnoses (2014).

In model 1, a higher adherence to the EAT-Lancet diet measured with the Knuppel score, the Hanley-Cook score, the Kesse-Guyot score, the Stubbendorff score, and the Bui score was associated with lower risk of all-cause dementia by 2014 (Table 2). After adding education as a potential confounder, evidence of associations with the Knuppel score (HR: 0.94 [95 % CI: 0.89, 0.99]), the Kesse-Guyot score (HR: 0.91 [95 % CI: 0.85, 0.97]), and the Bui score (HR: 0.92 [95 % CI: 0.86, 0.99]) remained statistically significant. After additional adjustment in model 2, only the association between the Kesse-Guyot score and incident dementia remained (HR: 0.93 [95 % CI: 0.87, 0.99]) (Table 2, and Fig. 2). The associations were similar when adherence to the EAT-Lancet diet was investigated in relation to all-cause dementia until 2020 (Table 2, and Fig. 2). The associations also remained similar when participants in Q5 was compared to participants in Q1. There was evidence of associations between the EAT-Lancet diet and all-cause dementia measured with the Kesse-Guyot score (HR: 0.82 [95 % CI: 0.70, 0.96]), and the Hanley-Cook score (HR: 0.79 [95 % CI: 0.65, 0.95]) (supplementary Table 2).

**Table 2:** Associations between the EAT-Lancet diet and all-cause dementia.

	Dementia (2014) *n* = 1,783 (1,773)^a^				Dementia (2020) *n* = 2,976 (2,963)^a^			
*N* = 25,898	Model 1		Model 2^a^		Model 1		Model 2^a^	
	HR (95 % CI)	P-value	HR (95 % CI)	P-value	HR (95 % CI)	P-value	HR (95 % CI)	P-value
Knuppel	0.93 (0.88, 0.98)	0.012	0.95 (0.90, 1.01)	0.073	0.95 (0.91, 0.99)	0.012	0.96 (0.92, 1.01)	0.089
Trijsburg	0.96 (0.91, 1.00)	0.052	0.97 (0.93, 1.02)	0.21	0.98 (0.94, 1.01)	0.15	0.99 (0.96, 1.03)	0.55
Hanley-Cook	0.95 (0.90, 0.99)	0.022	0.96 (0.91, 1.00)	0.069	0.96 (0.93, 0.99)	0.023	0.97 (0.93, 1.00)	0.083
Kesse-Guyot	0.89 (0.84, 0.95)	0.00026	0.93 (0.87, 0.99)	0.019	0.91 (0.87, 0.96)	0.00026	0.94 (0.90, 0.99)	0.025
Stubbendorff	0.94 (0.88, 1.00)	0.049	0.97 (0.91, 1.03)	0.32	0.96 (0.91, 1.01)	0.10	0.99 (0.94, 1.04)	0.65
Colizzi	0.95 (0.90, 1.00)	0.057	0.97 (0.92, 1.02)	0.23	0.96 (0.92, 1.00)	0.043	0.98 (0.93, 1.02)	0.24
Bui	0.90 (0.83, 0.96)	0.0031	0.93 (0.87, 1.01)	0.083	0.94 (0.89, 0.99)	0.022	0.98 (0.92, 1.03)	0.41

*Note:* Associations between the EAT-Lancet diet measured with seven EAT-Lancet diet scores and all-cause dementia (yes/no) by 2014 (validated register data), and 2020 (validated register diagnoses until 2014, and unvalidated diagnoses between 2015 and 2020) were analysed with Cox proportional hazard models. Model 1 was adjusted for age, sex, season, diet method version, and energy intake. Model 2 was adjusted for age, sex, season, dietary method version, energy intake, education, smoking, alcohol consumption, physical activity, and BMI. Results are presented per 10 % in increment scores. The scores were labelled based on first authors of the articles that reported the construction of the scores.

a There were missing data on smoking (*n* = 8), BMI (*n* = 40), and physical activity (*n* = 120). In total 0.64 % (*n* = 165) of cases were excluded in model 2. Out of the participants with missing data, there were 10 participants with a dementia diagnosis in 2014, and 13 participants in 2020.

### 3.4 Associations between the EAT-Lancet diet and Alzheimer's disease

In model 1, a higher adherence to the EAT-Lancet diet measured with the Kesse-Guyot score was associated with lower risk of AD (Table 2). This association remained when education was added as a confounder (HR: 0.89 [95 % CI: 0.82, 0.96]), and after additional adjustment in model 2 (HR: 0.87 [95 % CI: 0.80, 0.95]) (Table 3, Fig. 2). No associations were found between the EAT-Lancet diet and AD with the other scores (Table 3, Fig. 2). The associations remained similar when participants in Q5 were compared to participants in Q1, with a significant association between the EAT-Lancet diet measured with the Kesse-Guyot score and a lower risk of AD (HR: 0.73 [95 % CI: 0.59, 0.90]) (supplementary Table 3).

**Table 3:** Associations of the EAT-Lancet diet with Alzheimer's disease and vascular dementia.

	Alzheimer's disease *n* = 1,040 (1,034)^a^				Vascular dementia *n* = 426 (423)^a^			
*N* = 25,898	Model 1		Model 2		Model 1		Model 2	
	HR (95 % CI)	P-value	HR (95 % CI)	P-value	HR (95 % CI)	P-value	HR (95 % CI)	P-value
Knuppel	0.96 (0.89, 1.03)	0.25	0.96 (0.89, 1.03)	0.26	0.92 (0.82, 1.03)	0.13	0.97 (0.86, 1.09)	0.62
Trijsburg	0.98 (0.93, 1.04)	0.54	0.98 (0.92, 1.04)	0.51	0.94 (0.86, 1.03)	0.18	0.99 (0.90, 1.09)	0.86
Hanley-Cook	0.97 (0.91, 1.03)	0.29	0.97 (0.91, 1.03)	0.30	0.98 (0.89, 1.07)	0.63	1.01 (0.92, 1.11)	0.89
Kesse-Guyot	0.88 (0.81, 0.95)	0.0015	0.87 (0.80, 0.95)	0.0020	0.86 (0.76, 0.98)	0.020	0.96 (0.84, 1.10)	0.54
Stubbendorff	0.98 (0.90, 1.07)	0.68	0.98 (0.90, 1.07)	0.66	0.87 (0.77, 0.99)	0.043	0.96 (0.84, 1.10)	0.53
Colizzi	0.96 (0.90, 1.03)	0.30	0.96 (0.89, 1.03)	0.26	0.93 (0.83, 1.04)	0.18	0.99 (0.89, 1.11)	0.89
Bui	0.94 (0.85, 1.04)	0.21	0.93 (0.84, 1.03)	0.19	0.82 (0.71, 0.96)	0.011	0.94 (0.81, 1.10)	0.45

*Note:* Associations between the EAT-Lancet diet measured with seven EAT-Lancet scores and Alzheimer's disease (pure, and with vascular pathology), and vascular dementia (yes/no, until 2014) were analysed with Cox proportional hazard models. Model 1 was adjusted for age, sex, season, diet method version, and energy intake. Model 2 was adjusted for age, sex, season, dietary method version, energy intake, education, smoking, alcohol consumption, physical activity, and BMI. Results are presented per 10 % in increment scores. The scores were labelled based on first authors of the articles that reported the construction of the scores.

a There were missing data on smoking (*n* = 8), BMI (*n* = 40), and physical activity (*n* = 120). In total 0.64 % (*n* = 165) of cases were excluded in model 2. Out of these, there were 6 participants with Alzheimer's disease excluded, and 3 participants with vascular dementia excluded.

### 3.5 Associations between the EAT-Lancet diet and vascular dementia

In model 1, a higher adherence measured with the Kesse-Guyot score (HR: 0.86 [95 % CI: 0.76, 0.98]), the Stubbendorff score (HR: 0.87 [95 % CI: 0.77, 0.99]), and the Bui score (HR: 0.82 [95 % CI: 0.71, 0.96]) was associated with lower risk of vascular dementia (Table 3). However, these associations did not remain when education was added as a confounder, or after additional adjustment in model 2 (Table 3, Fig. 2). No other associations between the EAT-Lancet diet and VaD were detected (Table 3, Fig. 2, supplementary Table 4).

### 3.6 Interaction analyses

Evidence of an interaction (*p*: <0.10) between the EAT-Lancet diet and *APOE* ε4 status (carrier/non-carrier) was found when adherence was measured with the Knuppel score (*p*: 0.026), the Hanley-Cook score (*p*: 0.013), the Kesse-Guyot score (*p*: 0.076), and the Bui score (*p*: 0.062) in relation to all-cause dementia 2014 (supplementary Table 5), and with the Knuppel score (*p*: 0.049), and the Hanley-Cook score (*p*: 0.012) in relation to AD (supplementary Table 6). Among *APOE* ε4 non-carriers, a higher adherence measured with the Knuppel score (HR: 0.89, 95 % CI: 0.82, 0.96), the Hanley-Cook score (HR: 0.91, 95 % CI: 0.85, 0.97), the Kesse-Guyot score (HR: 0.87, 95 % CI: 0.80, 0.96), the Bui score (HR: 0.86, 95 % CI: 0.78, 0.96), and the Colizzi score (HR: 0.92, 95 % CI: 0.85, 0.99) was associated with lower risk of all-cause dementia (supplementary Table 5, Fig. 2). Moreover, a higher adherence measured with the Knuppel score (HR: 0.87, 95 % CI: 0.78, 0.98), the Hanley-Cook score (HR: 0.88, 95 % CI: 0.80, 0.97), and the Kesse-Guyot score (HR: 0.81, 95 % CI: 0.71, 0.92) was associated with lower risk of AD among *APOE* ε4 non-carriers (supplementary Table 6, Fig. 2). There was no evidence of an association between the EAT-Lancet diet and AD or all-cause dementia among *APOE* ε4 carriers (supplementary Table 5 and 6, Fig. 2). No evidence of an interaction between the EAT-Lancet diet and *APOE* ε4 status was found in relation to VaD (supplementary Table 7, Fig. 2).

### 3.7 Additional analyses

There was no evidence of an association between any EAT-Lancet diet score and Aβ42 pathology (supplementary Table 8).

There was no evidence of differences in estimates between the EAT-Lancet diet scores in relation to dementia when pairwise comparisons were performed, expressed as log HR differences visualized in heatmaps with significance markers in supplementary figure 4.

### 3.8 Sensitivity analyses

Excluding BMI as a confounder in model 2 did not influence the results (supplementary Table 9). Excluding participants with either diabetes (19 % of total participants, and 11 % of dementia cases), or incident dementia <5 years from baseline (4 % of dementia cases) showed similar results (supplementary Tables 10–19). The association between the Kesse-Guyot score and AD remained statistically significant, while the association with all-cause dementia did not (supplementary Tables 10, 11, 15, 16). Moreover, the interaction between *APOE* ε4*EAT-Lancet diet in relation to all-cause dementia remained with the Knuppel score and the Hanley-Cook score (supplementary Tables 12, and 17), and with the Hanley-Cook score in relation to AD (supplementary Table 13, and 18). Furthermore, the association between higher adherence to the EAT-Lancet diet and reduced risk of all-cause dementia among *APOE* ε4 non-carriers remained with the Knuppel score, the Hanley-Cook score, and the Bui score (supplementary Table 12, and 17), and with the Kesse-Guyot score in relation to AD (supplementary Table 13, and 18). Additionally, the association between the Kesse-Guyot score and all-cause dementia remained statistically significant among *APOE* ε4 non-carriers in the analyses where participants with incident dementia <5 years from baseline were excluded (supplementary Table 17), but not in the analyses where participants with diabetes were excluded (supplementary Table 12).

No associations were detected between any EAT-Lancet diet scores and VaD in the sensitivity analyses (supplementary Tables 9, 14, 16, and 19).

## 4 Discussion

In this population-based study with a mean follow-up time of 18 years, we found an association between higher adherence to the EAT-Lancet diet and a reduced risk of AD and all-cause dementia with one out of seven EAT-Lancet diet scores. Additionally, the study suggests a potential interaction between the EAT-Lancet diet and *APOE* ε4 status in relation to AD and all-cause dementia, with a risk-reducing effect observed with several of the scores among *APOE* ε4 non-carriers, but not among *APOE* ε4 carriers. No association was observed between the EAT-Lancet diet and incident VaD.

Few other studies have investigated the impact of the EAT-Lancet diet on cognitive decline and dementia. Similar to our study, previous studies reported either no associations, or a risk reducing effect with a higher adherence to the diet [13,31,32]. In the UK biobank study, they found that higher adherence to the EAT-Lancet diet (Knuppel score) was associated with lower risk of all-cause dementia among participants with high socioeconomic standard, but no association was detected among those with low socioeconomic standard [13]. A Brazilian study found that a higher adherence to the EAT-Lancet diet (score by Cacau et al. 2021) was associated with slower memory decline among participants over the age of 60 years, and with slower global cognitive decline among high-income participants, but not among low-income participants [31]. A third study found that higher adherence to the EAT-Lancet diet (modified Stubbendorff score), was associated with better global cognitive function among cognitively healthy older adults [32].

The results from our study support the notion that there may be an interplay between *APOE* ε4 status and diet in relation to AD and dementia [33]. *APOE* ε4 is associated with AD pathology, specifically with an earlier start of Aβ accumulation, which is the starting point of AD. Individuals with AD who are *APOE* ε4 carriers begin accumulating Aβ earlier than non-carriers [34]. However, much less is known about the causes of Aβ accumulation in non-ε4 carriers with AD. Based on our findings, a possible hypothesis is that environmental factors, such as diet, may play a more significant role in these individuals compared to the genetic risk observed in ε4 carriers with AD. Nonetheless, we could not find any association between EAT-Lancet scores and CSF Aβ42 in the smaller subsample with available CSF data. If there is a true association between the EAT-Lancet diet and AD, it could potentially thus be mediated by other factors than Aβ, but we found no support that this would be through vascular factors since no robust association with VaD was found. Similar to previous studies, our findings indicate that the protective effects of greater adherence to a healthy diet are evident only in individuals with a low genetic predisposition to AD [[35], [36], [37], [38]]. However, results from previous research are inconsistent. A previous study investigating the impact of both a healthy and a western-like dietary pattern in relation to all-cause dementia, found a risk reducing effect of a healthy dietary pattern among *APOE* ε4 non-carriers, and a risk increasing effect of a western dietary pattern among *APOE* ε4 carriers [30]. Thus, indicating that diet may impact the risk among both carriers and non-carriers, but that the impact may differ depending on food combinations. While mechanistic pathways linking diet with AD and dementia are poorly understood, it has been suggested that cardio- and cerebrovascular mechanisms (e.g., lipid and glucose metabolism) may be involved, and that healthy dietary patterns could have anti-inflammatory properties [33]. We can only speculate on why protective effects was seen only among ε4 non-carriers. Potentially, *APOE* ε4 carriership may intervene on pathways linking diet with dementia (e.g., cholesterol metabolism), attenuating the protective effects of a healthy diet [39]. However, adhering to a healthy diet may still be beneficial for ε4 carriers, as a previous study found that a higher adherence to a western diet increased the risk of dementia among ε4 carriers [30]. Even though all the EAT-Lancet diet scores included in our study were constructed to measure the same reference diet, the scores interpret the diet differently, which may explain the inconsistencies in results. For example, a high score measured with the Knuppel score could reflect a diet high in vegetables, fruits and nuts, while a low score could reflect a diet high in foods that are promoted in the EAT-Lancet diet, such as fish and legumes [12,16]. The Bui and the Hanley-Cook score could deviate from the reference diet, as the Bui score had higher cutoff values for foods to limit than the reference diet (e.g., allowing a higher intake of animal-derived foods), and the Hanley-Cook score included minimum limit cutoffs that were not in line with the EAT-Lancet reference diet (e.g., provided a lower score among those with 0 *g* intake of red meat and dairy) [21,24]. The Kesse-Guyot score had the widest score-range, and a high score could be due to a high intake of foods to promote, low intake of foods to limit, or a combination, according to the score definitions. Furthermore, the Kesse-Guyot score was adjusted for energy intake. Potentially, this may explain the more robust association with the Kesse-Guyot score. However, the direction of associations was similar for all scores in relation to AD and all-cause dementia. The associations between the EAT-Lancet diet and incident dementia were attenuated when participants with either diabetes or incident dementia <5 years from baseline were excluded, which may be explained by potential changes in dietary intake due to diabetes or preclinical/prodromal stages of dementia. However, participants with dementia at baseline year were excluded in all analyses, and diabetes may be a potential pathway linking diet with dementia [2].

A previous study including the same sample as our study, found no associations between the MeDi and incident dementia [19]. Even though there are similarities between the MeDi and the EAT-Lancet diet (e.g., emphasising plant-based foods), the MeDi was not designed to achieve global equity in access to healthy food, improve food production and environmental sustainability as the EAT-Lancet diet is [6]. Future studies are needed to investigate potential differences between the impact of the MeDi and the EAT-Lancet diet in relation to incident dementia.

### 4.1 Strengths and limitations

There were several strengths of this study, such as the prospective study design, the large sample size, the long follow-up time, the comprehensive dietary examination, and the validated dementia diagnoses by trained physicians. Another strength was the inclusion of seven different scores to measure adherence to the EAT-Lancet reference diet. Including seven different scoring systems increases the ability to compare results with other studies investigating associations between the EAT-Lancet diet and dementia. Furthermore, by including several scores, we could explore the robustness of associations. The sensitivity analysis including unvalidated dementia diagnosis between 2015 and 2020 is another strength, as a previous study showed that the general dementia diagnosis (all-cause dementia) until 2014 was valid in 96 % of the cases [40]. We did not include AD and VaD data between 2015 and 2020, as 40 % of the specific dementia diagnoses until 2014 were changed during the re-evaluation process [40]. Some limitations need to be addressed. As in all observational studies, we cannot rule out the possibility of residual confounding. Additionally, we cannot rule out the possibility that there may be some misreporting of dietary intake, and that participants may have changed their dietary intake during the study period. However, previous validation studies indicate that the quality of the dietary data is high in the MDCS [7,41]. Furthermore, previous studies have shown acceptable agreement between repeated dietary measures among participants of similar age as in our study [42,43]. The disease progression in AD can start as early as 20 years before the first symptoms appear [2]. Thus, a strength of this study is that the impact of dietary habits earlier in life could be explored. This is especially important as dietary habits may change in the preclinical phase due to the disease progression (e.g., weight loss is a common early sign of dementia).

## 5 Conclusion

The environmentally sustainable EAT-Lancet diet had no negative effect on dementia risk across different scoring methods. The results indicated a reduced risk of AD and all-cause dementia among *APOE* ε4 non-carriers, but not among *APOE* ε4 carriers. These findings can inform future research exploring the EAT-Lancet diet in relation to cognitive decline and dementia. Future studies should consider the potentially modifying effect of *APOE* ε4 status in this context and consider the implications of methodological differences in measuring adherence to the EAT-Lancet diet. While intervention studies are needed to further clarify the impact of the EAT-Lancet diet on dementia incidence, results from this study indicate that environmental sustainability can be implemented into dietary guidelines in dementia prevention strategies.

## Data availability

Data described in the manuscript can be made available upon request pending application and approval by the chair of the steering committee for the cohort, in accordance with General Data Protection Regulation (GDPR) rules.

## Ethical standards

The ethics committee at Lund University approved the study (LU 51–90). The participants gave written informed consent to participate in the study, and for their data to be used for research purposes, according to the Helsinki declaration.

## Declaration of generative AI and AI-assisted technologies in the writing process

We have not used AI in the writing process.

## Funding

The Dementia foundation, Handlanden Hjalmar Svenssons research fund, the Swedish Research Council (2018-02052), the Crafoord foundation (20230771), the Påhlsson Foundation and Agenda 2030 Graduate School, Lund University.

## CRediT authorship contribution statement

**Jessica Samuelsson:** Writing – original draft, Methodology, Funding acquisition, Formal analysis, Conceptualization. **Isabelle Glans:** Writing – review & editing, Methodology, Data curation, Conceptualization. **Anna Stubbendorff:** Writing – review & editing, Methodology, Data curation, Conceptualization. **Ulrika Ericson:** Writing – review & editing, Methodology, Data curation. **Sebastian Palmqvist:** Writing – review & editing, Methodology, Funding acquisition, Data curation. **Oskar Hansson:** Writing – review & editing, Methodology, Data curation. **Emily Sonestedt:** Writing – review & editing, Methodology, Funding acquisition, Data curation, Conceptualization.

## Declaration of competing interest

The authors declare the following financial interests/personal relationships which may be considered as potential competing interests: Sebastian Palmqvist reports a relationship with Bioartic, Biogen, Esai, Eli Lilly, Novo Nordisk, and Roche that includes: consulting or advisory and speaking and lecture fees. Oskar Hansson reports a relationship with Alzpath, BioArctic, Biogen, Bristol Meyer Squibb, Eisai, Eli Lilly, Fujirebio, Merck, Novartis, Novo Nordisk, Roche, Sanofi and Siemens that includes: consulting or advisory and speaking and lecture fees. If there are other authors, they declare that they have no known competing financial interests or personal relationships that could have appeared to influence the work reported in this paper.

## Appendix Supplementary materials

Image, application 1
