# Supplementary material for: Associations between the EAT-Lancet planetary health diet and incident dementia
Source: J Prev Alzheimers Dis. 2025 Apr 12;12(6):100166. doi: 10.1016/j.tjpad.2025.100166 (PMC12434276; doi:10.1016/j.tjpad.2025.100166)
Supplement: Supplementary file 1 [file mmc1.docx]

**Supplemental data**

**Table of contents:**

| Supplementary figure 1 | Overview of food group categorisation for the EAT–Lancet diet scores................... | 3 |
| --- | --- | --- |
| Supplementary figure 2 | Boxplots of adherence to the EAT-Lancet diet measured with seven different scores……………………………………………………………………………… | 4 |
| Supplementary figure 3 | Correlation matrix of the seven EAT-Lancet diet scores..………………………… | 5 |
| Supplementary figure 4 | Differences in HRs between seven EAT-Lancet diet scores and incident dementia.. | 6 |
| Supplementary table 1 | Description of seven EAT-Lancet diet scores ……………………………………… | 7 |
| Supplementary table 2 | Comparisons of Q5 to Q1 for each of the EAT-Lancet diet scores in relation to all-cause dementia. …………………………………………………………………….. | 8 |
| Supplementary table 3 | Comparisons of Q5 to Q1 for each of the EAT-Lancet diet scores in relation to Alzheimer’s disease………………………………………………………………… | 8 |
| Supplementary table 4 | Comparisons of Q5 to Q1 for each of the EAT-Lancet diet scores in relation to vascular dementia…………………………………………………………………… | 8 |
| Supplementary table 5 | Associations between the EAT-Lancet diet and all-cause dementia by *APOE* ε4 status………………………………………………………………………………… | 9 |
| Supplementary table 6 | Associations between the EAT-Lancet diet and Alzheimer’s disease by *APOE* ε4 status. ………………………………………….......................................................... | 10 |
| Supplementary table 7 | Associations between the EAT-Lancet diet and vascular dementia by *APOE* ε4 status..………………………………………….......................................................... | 11 |
| Supplementary table 8 | Associations between the EAT-Lancet diet and Aβ42 pathology. ………………………………………………………………………………………. | 12 |
| Supplementary table 9 | Associations between the EAT-Lancet diet and incident dementia without including BMI as a potential confounder.……………………………....................... | 12 |
| Supplementary table 10 | Associations between the EAT-Lancet diet and all-cause dementia when participants with diabetes were excluded.……………………………....................... | 13 |
| Supplementary table 11 | Associations of the EAT-Lancet diet with Alzheimer’s disease and vascular dementia when participants with diabetes were excluded..………………………… | 13 |
| Supplementary table 12 | Associations between the EAT-Lancet diet and all-cause dementia by *APOE* ε4 status when participants with diabetes were excluded.……………………………... | 14 |
| Supplementary table 13 | Associations between the EAT-Lancet diet and Alzheimer’s disease by *APOE* ε4 status when participants with diabetes were excluded.…………………..…………. | 15 |
| Supplementary table 14 | Associations between the EAT-Lancet diet and vascular dementia by *APOE* ε4 status when participants with diabetes were excluded.……………………………... | 16 |
| Supplementary table 15 | Associations between the EAT-Lancet diet and all-cause dementia when participants with incident dementia <5 years from baseline were excluded ……….. | 17 |
| Supplementary table 16 | Associations of the EAT-Lancet diet with Alzheimer’s disease and vascular dementia when participants with incident dementia <5 years from baseline were excluded..………………………………………………………………………….... | 17 |
| Supplementary table 17 | Associations between the EAT-Lancet diet and all-cause dementia by *APOE* ε4 status when participants with incident dementia <5 years from baseline were excluded..………………………………………………………………………….... | 18 |
| Supplementary table 18 | Associations between the EAT-Lancet diet and Alzheimer’s disease by *APOE* ε4 status when participants with incident dementia <5 years from baseline were excluded.……………………………………………………………………………. | 19 |
| Supplementary table 19 | Associations between the EAT-Lancet diet and vascular dementia by *APOE* ε4 status when participants with incident dementia <5 years from baseline were excluded………………………………………………….…........................... | 20 |


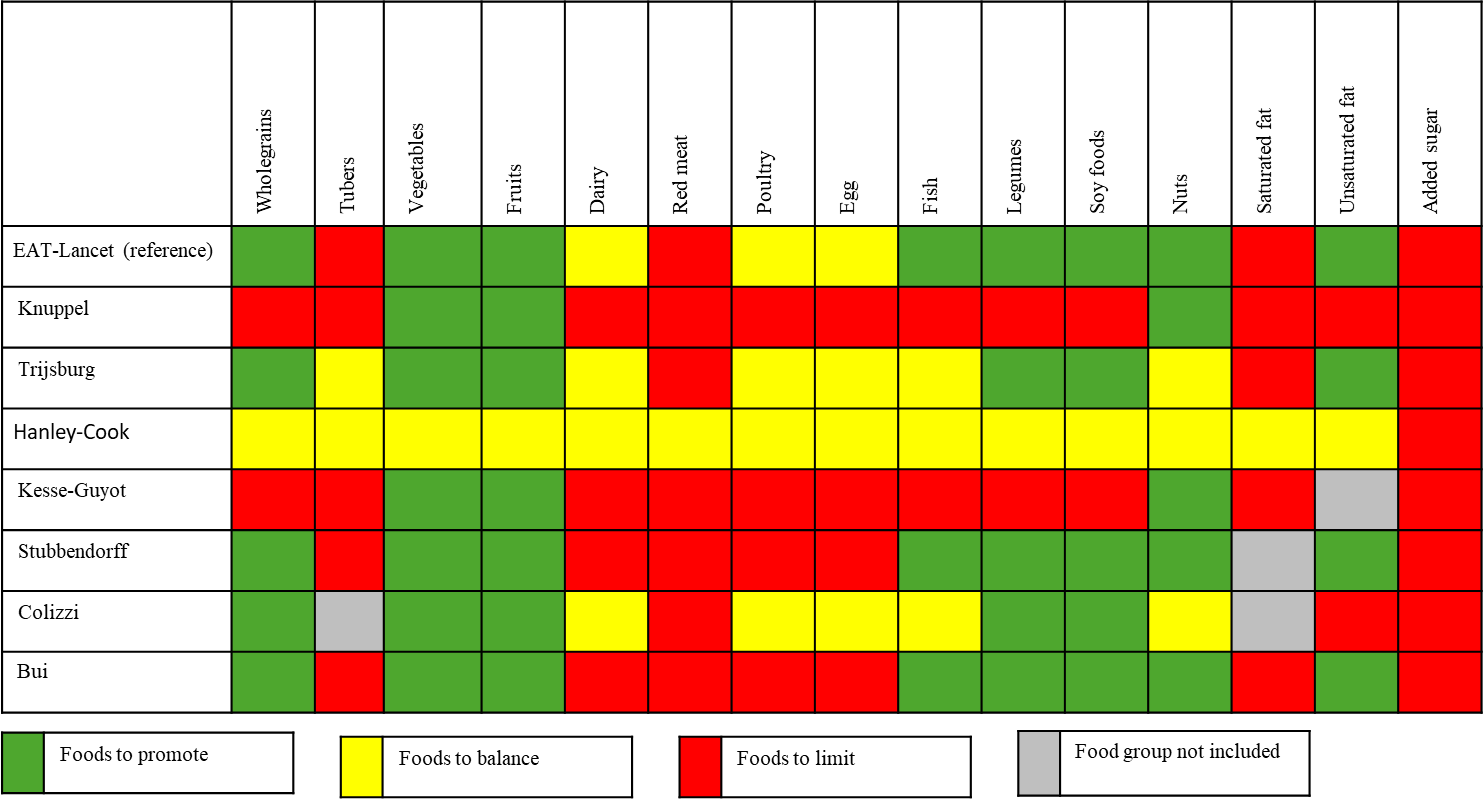
**Supplementary figure 1.** **Overview of food group categorisation for the EAT–Lancet diet scores.** The scores were labelled based on first authors of the articles reporting the construction of the scores. The interpretation to promote, balance, or limit the food groups was based on definitions in the EAT–Lancet reference diet reported by Willett et al. 2019


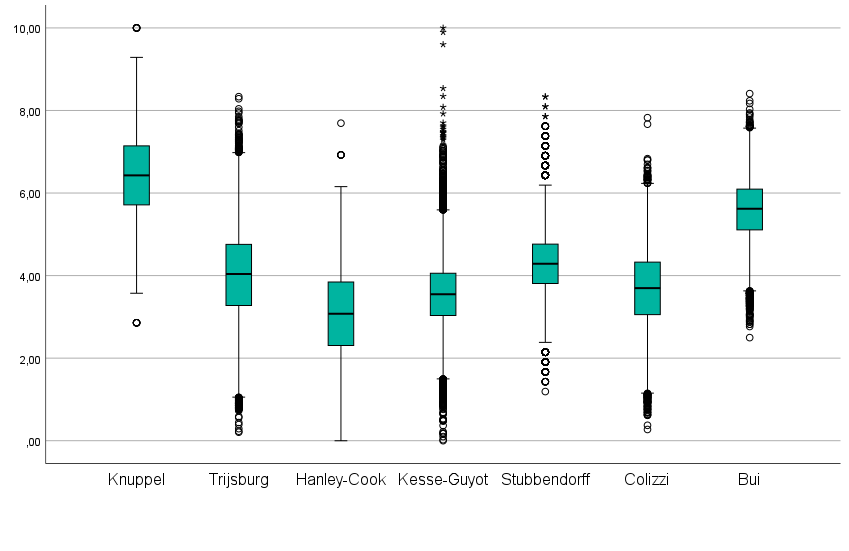
**Supplementary figure 2. Boxplots of adherence to the EAT-Lancet diet measured with seven different scores.** Adherence was reported per 10% in increment in score for comparability among participants with dietary and dementia data. The scores were labelled based on first authors of the articles reporting the construction of the scores.


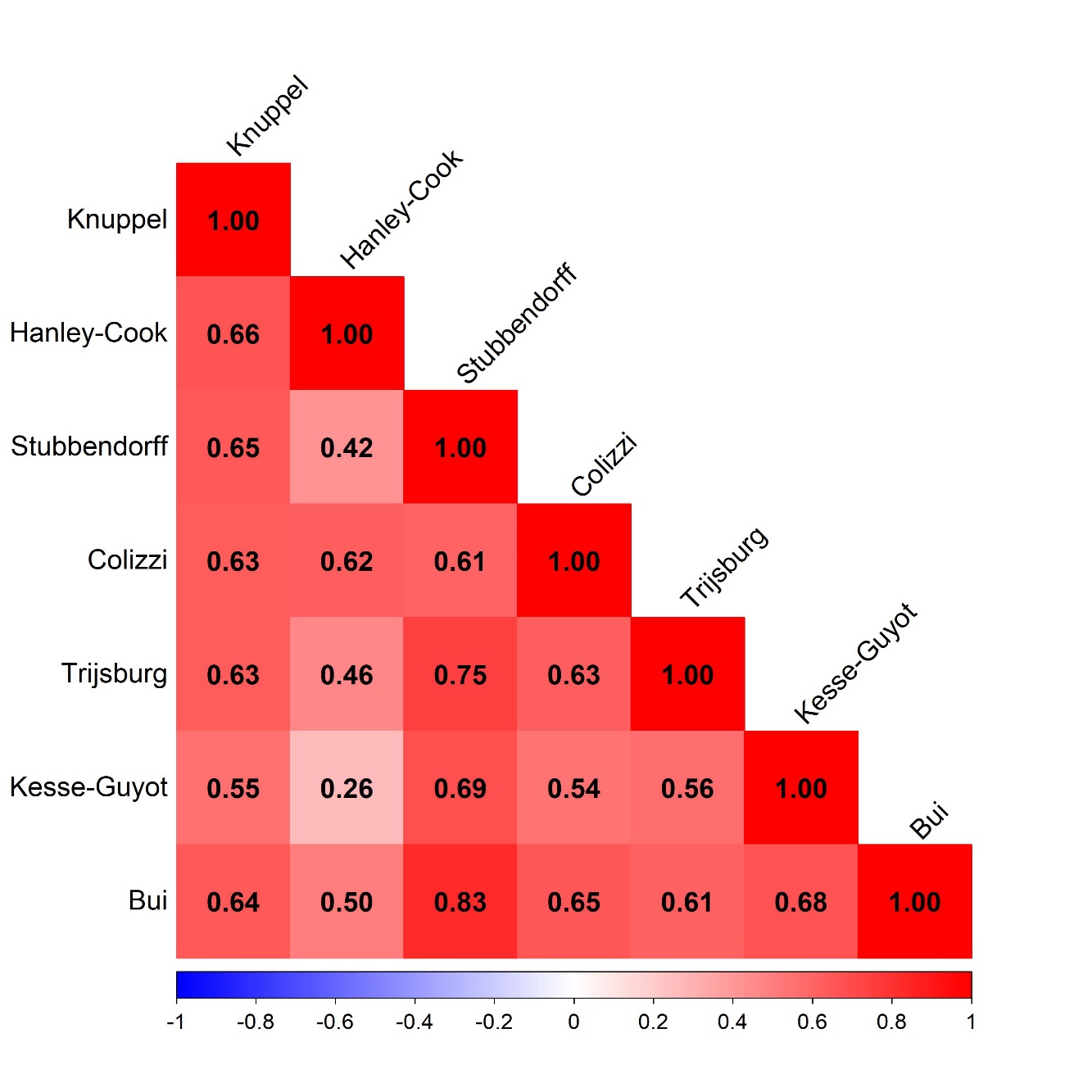
**Supplementary figure 3. Correlation matrix of the seven EAT-Lancet diet scores.** Pearson correlation coefficient (r) values are presented. All correlations were significant (*p* <0.001).


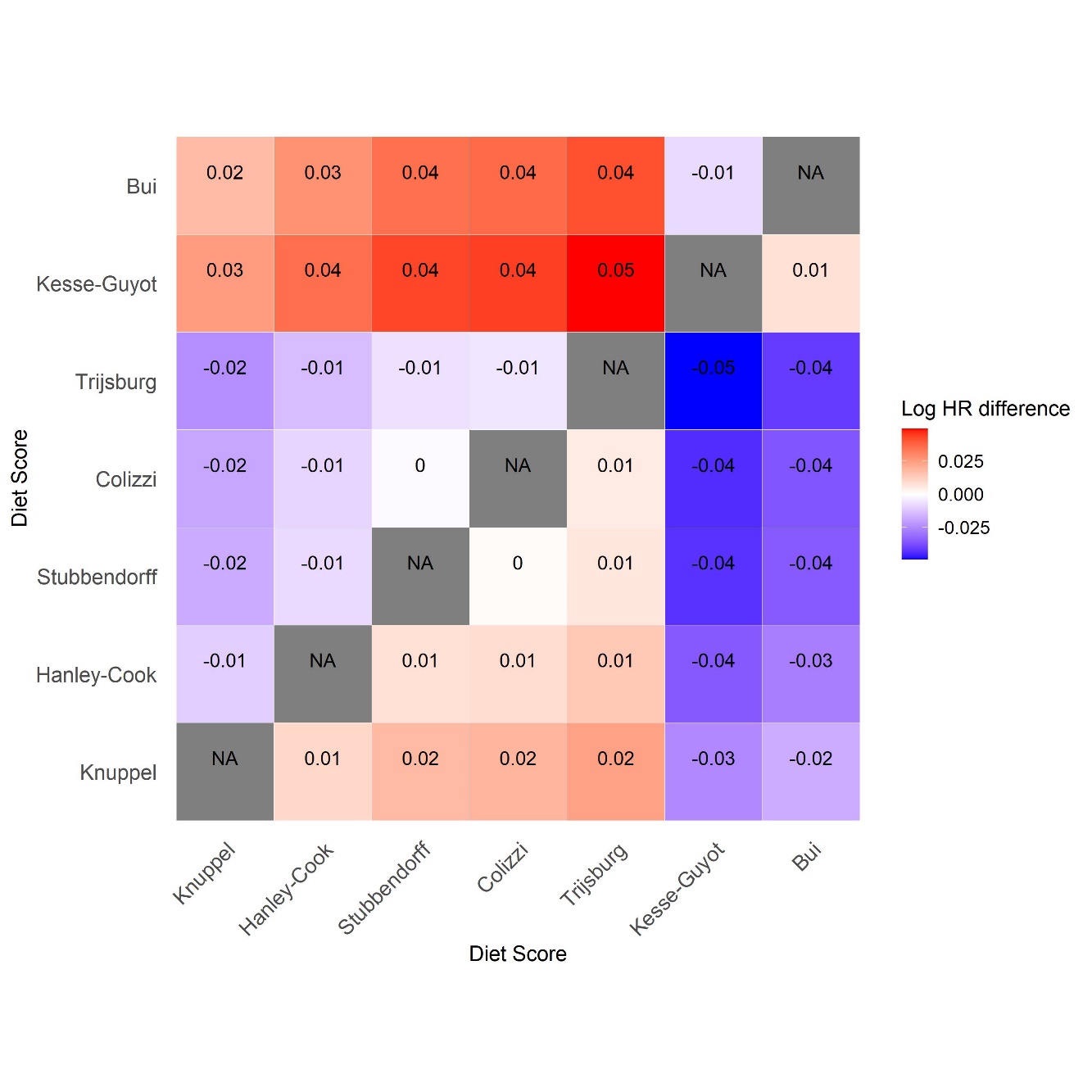
**Supplementary figure 4. Differences in HRs between seven EAT-Lancet diet scores and incident dementia.** We conducted a non-parametric bootstrap analysis (1,000 resamples) to compare the effects of seven dietary scores on incident dementia (2014) using Cox proportional hazards models adjusted for potential confounders. For each bootstrap iteration, log hazard ratios (log HRs) were estimated for all scores. Pairwise differences in log HRs were computed, and significance was assessed using two-sided bootstrap p-values. Differences were expressed as log HR differences and visualized in heatmaps with significance markers (p <0.05 = *, p <0.01 = **, p <0.001 = ***). No significance markers (*) = no significant differences in HR (95% CI) between the scores.

**Supplementary table 1. Description of seven EAT-Lancet diet scores**

|  | **Score name** | **Number dietary components (no. positive, balanced, negative)^a^** | **Type of score** | **Score description** | **Score range** |
| --- | --- | --- | --- | --- | --- |
| Knuppel et al. (2019) | EAT-Lancet diet score | 14 (3, 1, 10) | Binary score | 1 point if intake is above or below a set intake threshold, 0 if not. Intakes are based on g/day. The total score is the sum of points for each component. | 0-14 |
| Trijsburg et al. (2020) | World index for Sustainability and health | 13 (4, 9, 0) | Proportional score | 0 points if intake is below a lower limit for emphasised foods or above a limit for balanced and de-emphasised foods. Score of 10 within optimal range for de-emphasised and balanced foods, or above optimal threshold for emphasised foods. Intakes are based on g/day. | 0-130 |
| Hanley-Cook et al. (2021) | EAT–Lancet diet score with minimum intake values | 14 (0, 13, 1) | Binary score | 1 point for intake within the recommended range, 0 if not. Intakes are based on g/day. The total score is the sum of points for each component. | 0-14 |
| Kesse-Guyot et al. (2021) | EAT–Lancet diet index | 14 (3, 0, 11) | Proportional score | Based on cutoffs for each component (g/day), The index was computed by: {100 x [sum of all components] (a_i_ x (cutoff – [consumption x 2500/energy intake_j_])/(cutoff_i_)]))/14, where _i_ refers to one of the 14 food groups, _j_ is the individual, and a_i_ is 1 for components to limit and -1 for components to promote. Each food has a specific cutoff. Intakes are based on g/day | No set range |
| Stubbendorff et al. (2022) | EAT–Lancet index | 14 (7, 0, 7) | Ordinal score | Between 0 and 3 points according to level of adherence to the food component. The total score is the sum of points for each component. | 0-42 |
| Colizzi et al. (2023) | Healthy Reference Diet | 14 (5, 7, 2) | Proportional score | 0 points if no intake in emphasised foods or too high intake in de-emphasised foods. Proportional score up to 10 points up to optimal intake range. 10 points is within optimal range. For foods to balance, score proportional to upper and lower intake limits. Intakes are based on g/day. | 0-140 |
| Bui et al. (2024) | Planetary Health Diet Index | 15 (8, 0, 7) | Proportional score | The minimum score for each food group (0) was based on the consumption per day that reflects the least beneficial health effect of that food group (usually 0 g/d for healthy food groups). The maximum score for each food group [usually 10, except for non-soy legumes, and soy foods (maximum 5)] was based on the consumption per day that reflected the greatest beneficial health effect of that food group (usually 0 g/d for unhealthy food groups). | 0-140 |

^a^A positive component is an emphasised food group in the diet and provides a higher score for higher intakes. A balanced component means that a high score is received when the intake is within a certain range. A negative component is a component that is de-emphasised in the diet, providing a lower score for greater intakes.

**Supplementary table 2. Comparisons of Q5 to Q1 for each of the EAT-Lancet diet scores in relation to all-cause dementia.**

| **N = 25,898** | **All-cause dementia (2014)** |  |
| --- | --- | --- |
| *EAT-Lancet scores* | *Q5 vs. reference Q1* |  |
|  | HR (95% CI) | *p*-value |
| **Knuppel** | 0.88 (0.71, 1.09) | 0.24 |
| **Trijsburg** | 0.88 (0.76, 1.03) | 0.12 |
| **Hanley-Cook** | 0.79 (0.65, 0.95) | 0.013 |
| **Kesse-Guyot** | 0.82 (0.70, 0.96) | 0.014 |
| **Stubbendorff** | 0.96 (0.80, 1.14) | 0.63 |
| **Colizzi** | 0.90 (0.77, 1.05) | 0.19 |
| **Bui** | 0.85 (0.72, 1.01) | 0.064 |

Note: HRs and 95% CI comparing Q5 to Q1 for each of the EAT-Lancet diet scores in relation to all-cause dementia (2014). Analyses were performed with Cox proportional hazard models adjusted for age, sex, season, dietary method version, energy intake, education, smoking, alcohol consumption, physical activity, and BMI. Results are presented per 10% in increment scores. The scores were labelled based on first authors of the articles that reported the construction of the scores. There were missing data on smoking (n = 8), BMI (n = 40), and physical activity (n = 120). In total 0.64% (n = 165) of cases were excluded.

**Supplementary table 3. Comparisons of Q5 to Q1 for each of the EAT-Lancet diet scores in relation to Alzheimer’s disease.**

| **N = 25,898** | **Alzheimer’s disease** |  |
| --- | --- | --- |
| *EAT-Lancet scores* | *Q5 vs. reference Q1* |  |
|  | HR (95% CI) | *p*-value |
| **Knuppel** | 0.83 (0.63, 1.09) | 0.18 |
| **Trijsburg** | 0.85 (0.70, 1.04) | 0.11 |
| **Hanley-Cook** | 0.85 (0.70, 1.09) | 0.21 |
| **Kesse-Guyot** | 0.73 (0.59, 0.90) | 0.004 |
| **Stubbendorff** | 0.97 (0.76, 1.24) | 0.82 |
| **Colizzi** | 0.84 (0.69, 1.03) | 0.10 |
| **Bui** | 0.90 (0.72, 1.12) | 0.34 |

Note: HRs and 95% CI comparing Q5 to Q1 for each of the EAT-Lancet diet scores in relation to Alzheimer’s disease. Analyses were performed with Cox proportional hazard models adjusted for age, sex, season, dietary method version, energy intake, education, smoking, alcohol consumption, physical activity, and BMI. Results are presented per 10% in increment scores. The scores were labelled based on first authors of the articles that reported the construction of the scores. There were missing data on smoking (n = 8), BMI (n = 40), and physical activity (n = 120). In total 0.64% (n = 165) of cases were excluded.

**Supplementary table 4. Comparisons of Q5 to Q1 for each of the EAT-Lancet diet scores in relation to vascular dementia.**

| **N = 25,898** | **Vascular dementia** |  |
| --- | --- | --- |
| *EAT-Lancet scores* | *Q5 vs. reference Q1* |  |
|  | HR (95% CI) | *p*-value |
| **Knuppel** | 1.08 (0.68, 1.71) | 0.74 |
| **Trijsburg** | 0.98 (0.70, 1.38) | 0.93 |
| **Hanley-Cook** | 0.86 (0.58, 1.30) | 0.47 |
| **Kesse-Guyot** | 0.93 (0.67, 1.29) | 0.67 |
| **Stubbendorff** | 1.04 (0.73, 1.50) | 0.82 |
| **Colizzi** | 1.02 (0.74, 1.41) | 0.89 |
| **Bui** | 0.76 (0.53, 1.08) | 0.12 |

Note: HRs and 95% CI comparing Q5 to Q1 for each of the EAT-Lancet diet scores in relation to vascular dementia. Analyses were performed with Cox proportional hazard models adjusted for age, sex, season, dietary method version, energy intake, education, smoking, alcohol consumption, physical activity, and BMI. Results are presented per 10% in increment scores. The scores were labelled based on first authors of the articles that reported the construction of the scores. There were missing data on smoking (n = 8), BMI (n = 40), and physical activity (n = 120). In total 0.64% (n = 165) of cases were excluded.

**Supplementary table 5. Associations between the EAT-Lancet diet and all-cause dementia by *APOE* ε4 status.**

|  | **All-cause dementia n = 1,728 (1,720)^a^** | | |  |  |  | |
| --- | --- | --- | --- | --- | --- | --- | --- |
| **N = 24,987^a^** | **Model 1** |  | **Model 2** | | | |  |
| ***APOE ε4**EAT-Lancet diet scores^b^** | HR (95% CI) | P-value | HR (95% CI) | | | P-value | |
| ***APOE ε4**Knuppel** | 1.12 (1.01, 1.24) | 0.026 | 1.12 (1.01, 1.24) | | | 0.026 | |
| *APOE ε4* carriers | 0.98 (0.91, 1.06) | 0.61 | 1.00 (0.92, 1.08) | | | 0.95 | |
| *APOE ε4* non-carriers | 0.87 (0.81, 0.94) | 0.00063 | 0.89 (0.82, 0.96) | | | 0.0032 | |
| ***APOE ε4**Trijsburg** | 1.03 (0.94, 1.12) | 0.55 | 1.02 (0.94, 1.12) | | | 0.62 | |
| *APOE ε4* carriers | 0.96 (0.90, 1.03) | 0.24 | 0.97 (0.91, 1.04) | | | 0.42 | |
| *APOE ε4* non-carriers | 0.94 (0.88, 0.99) | 0.046 | 0.95 (0.89, 1.02) | | | 0.14 | |
| ***APOE ε4**Hanley-Cook** | 1.12 (1.02, 1.23) | 0.016 | 1.12 (1.02, 1.23) | | | 0.013 | |
| *APOE ε4* carriers | 1.01 (0.94, 1.08) | 0.82 | 1.02 (0.95, 1.09) | | | 0.57 | |
| *APOE ε4* non-carriers | 0.90 (0.84, 0.96) | 0.0019 | 0.91 (0.85, 0.97) | | | 0.0042 | |
| ***APOE ε4**Kesse-Guyot** | 1.13 (1.00, 1.27) | 0.054 | 1.12 (0.99, 1.26) | | | 0.076 | |
| *APOE ε4* carriers | 0.94 (0.86, 1.03) | 0.18 | 0.97 (0.89, 1.06) | | | 0.56 | |
| *APOE ε4* non-carriers | 0.84 (0.76, 0.91) | <0.0001 | 0.87 (0.80, 0.96) | | | 0.0036 | |
| ***APOE ε4**Stubbendorff** | 1.10 (0.98, 1.25) | 0.12 | 1.09 (0.97, 1.24) | | | 0.15 | |
| *APOE ε4* carriers | 0.98 (0.89, 1.07) | 0.57 | 1.00 (0.91, 1.09) | | | 0.99 | |
| *APOE ε4* non-carriers | 0.88 (0.81, 0.97) | 0.0073 | 0.91 (0.83, 1.00) | | | 0.053 | |
| ***APOE ε4**Colizzi** | 1.09 (0.98, 1.21) | 0.13 | 1.08 (0.98, 1.20) | | | 0.13 | |
| *APOE ε4* carriers | 0.98 (0.91, 1.06) | 0.65 | 1.00 (0.93, 1.08) | | | 0.96 | |
| *APOE ε4* non-carriers | 0.90 (0.84, 0.98) | 0.011 | 0.92 (0.85, 0.99) | | | 0.036 | |
| ***APOE ε4**Bui** | 1.15 (1.00, 1.32) | 0.053 | 1.14 (1.00, 1.31) | | | 0.062 | |
| *APOE ε4* carriers | 0.95 (0.86, 1.05) | 0.32 | 0.99 (0.89, 1.09) | | | 0.80 | |
| *APOE ε4* non-carriers | 0.83 (0.75, 0.92) | 0.00030 | 0.86 (0.78, 0.96) | | | 0.0067 | |

Note: Interactions between seven EAT-Lancet diet scores and *APOE ε4* (yes/no) in relation to all-cause dementia by 2014 were investigated with Cox proportional hazard models. Model 1 was adjusted for age, sex, season, diet method version, and energy intake. Model 2 was adjusted for age, sex, season, dietary method version, energy intake, education, smoking, alcohol consumption, physical activity, and BMI. Results are presented per 10% in increment scores. The scores were labelled based on first authors of the articles that reported the construction of the scores.

^a^Out of the total study sample (n = 25,898), there were 24,987 individuals with data on *APOE ε4* status (17,444 ε4 non-carriers, 7,543 ε4 carriers). Out of the 1,720 with dementia, n = 879 were *APOE ε4* carriers and n = 841 were *APOE ε4* non-carriers. There were missing data on BMI (n = 37), and physical activity (n = 114).

**^b^**Associations between the EAT-Lancet diet and all-cause dementia are reported by *APOE ε4* status (carriers/non-carriers) based on results from the interaction analyses for each of the EAT-Lancet diet scores.

**Supplementary table 6. Associations between the EAT-Lancet diet and Alzheimer’s disease by *APOE* ε4 status.**

|  | **Alzheimer’s disease n = 1,012 (1,008)** | | |  |  |  |  |
| --- | --- | --- | --- | --- | --- | --- | --- |
| **N = 24,987^a^** | **Model 1** |  | **Model 2** | | |  | |
| ***APOE ε4**EAT-Lancet diet scores^b^** | HR (95% CI) | P-value | HR (95% CI) | | | P-value | |
| ***APOE ε4**Knuppel** | 1.14 (1.0, 1.30) | 0.068 | 1.15 (1.00, 1.32) | | | 0.049 | |
| *APOE ε4* carriers | 1.00 (0.91, 1.10) | 0.99 | 1.00 (0.92, 1.10) | | | 0.98 | |
| *APOE ε4* non-carriers | 0.88 (0.79, 0.99) | 0.027 | 0.87 (0.78, 0.98) | | | 0.018 | |
| ***APOE ε4**Trijsburg** | 1.04 (0.93, 1.17) | 0.49 | 1.05 (0.93, 1.18) | | | 0.46 | |
| *APOE ε4* carriers | 0.99 (0.92, 1.07) | 0.85 | 0.99 (0.92, 1.07) | | | 0.82 | |
| *APOE ε4* non-carriers | 0.95 (0.93, 1.17) | 0.30 | 0.95 (0.86, 1.04) | | | 0.26 | |
| ***APOE ε4**Hanley-Cook** | 1.15 (1.02, 1.31) | 0.022 | 1.17 (1.04, 1.33) | | | 0.012 | |
| *APOE ε4* carriers | 1.03 (0.95, 1.12) | 0.46 | 1.03 (0.96, 1.12) | | | 0.38 | |
| *APOE ε4* non-carriers | 0.89 (0.81, 0.98) | 0.020 | 0.88 (0.80, 0.97) | | | 0.013 | |
| ***APOE ε4**Kesse-Guyot** | 1.14 (0.97, 1.35) | 0.10 | 1.14 (0.97, 1.35) | | | 0.12 | |
| *APOE ε4* carriers | 0.92 (0.83, 1.02) | 0.13 | 0.92 (0.83, 1.02) | | | 0.12 | |
| *APOE ε4* non-carriers | 0.81 (0.71, 0.92) | 0.0011 | 0.81 (0.71, 0.92) | | | 0.0014 | |
| ***APOE ε4**Stubbendorff** | 1.10 (0.93, 1.29) | 0.27 | 1.11 (0.94, 1.31) | | | 0.23 | |
| *APOE ε4* carriers | 1.01 (0.91, 1.12) | 0.87 | 1.01 (0.91, 1.12) | | | 0.87 | |
| *APOE ε4* non-carriers | 0.92 (0.81, 1.05) | 0.21 | 0.91 (0.80, 1.04) | | | 0.17 | |
| ***APOE ε4**Colizzi** | 1.03 (0.90, 1.20) | 0.59 | 1.05 (0.91, 1.21) | | | 0.47 | |
| *APOE ε4* carriers | 0.97 (0.88, 1.06) | 0.48 | 0.97 (0.88, 1.06) | | | 0.48 | |
| *APOE ε4* non-carriers | 0.93 (0.83, 1.04) | 0.21 | 0.92 (0.82, 1.03) | | | 0.14 | |
| ***APOE ε4**Bui** | 1.10 (0.92, 1.33) | 0.30 | 1.12 (0.93, 1.35) | | | 0.24 | |
| *APOE ε4* carriers | 0.97 (0.85, 1.09) | 0.59 | 0.97 (0.86, 1.10) | | | 0.59 | |
| *APOE ε4* non-carriers | 0.88 (0.75, 1.02) | 0.080 | 0.86 (0.74, 1.01) | | | 0.060 | |

Note: Interactions between seven EAT-Lancet diet scores and *APOE ε4* (yes/no) in relation to Alzheimer’s disease (AD) were investigated with Cox proportional hazard models. Model 1 was adjusted for age, sex, season, diet method version, and energy intake. Model 2 was adjusted for age, sex, season, diet method version, energy intake, education, smoking, alcohol consumption, physical activity, and BMI. Results are presented per 10% in increment scores. The scores were labelled based on first authors of the articles that reported the construction of the scores.

^a^There were 24,987 individuals with data on *APOE* ε4 status (17,444 non-carriers, 7,543 carriers). Out of the 1,008 with AD, 608 were *APOE ε4* carriers and 400 were *APOE ε4* non-carriers. There were missing data on BMI (n = 37), and physical activity (n = 114).

**^b^**Associations between the EAT-Lancet diet and Alzheimer’s disease is reported by *APOE ε4* status (carriers/non-carriers) based on results from the interaction analyses for each of the EAT-Lancet diet scores.

**Supplementary table 7. Associations between the EAT-Lancet diet and vascular dementia by *APOE* ε4 status.**

|  | **Vascular dementia= 412 (409)** | | |  |  |  |  |
| --- | --- | --- | --- | --- | --- | --- | --- |
| **N = 24,987^a^** | **Model 1** |  | **Model 2** | | |  | |
| ***APOE ε4**EAT-Lancet diet scores^b^** | HR (95% CI) | P-value | HR (95% CI) | | | P-value | |
| ***APOE ε4**Knuppel** | 1.00 (0.81, 1.24) | 0.98 | 0.98 (0.79, 1.22) | | | 0.87 | |
| *APOE ε4* carriers | 0.92 (0.78, 1.04 | 0.39 | 0.96 (0.81, 1.15) | | | 0.69 | |
| *APOE ε4* non-carriers | 0.92 (0.80, 1.07) | 0.27 | 0.98 (0.85, 1.14) | | | 0.80 | |
| ***APOE ε4**Trijsburg** | 0.94 (0.78, 1.13) | 0.52 | 0.92 (0.76, 1.11) | | | 0.38 | |
| *APOE ε4* carriers | 0.90 (0.78, 1.04) | 0.16 | 0.93 (0.80, 1.08) | | | 0.37 | |
| *APOE ε4* non-carriers | 0.96 (0.85, 1.07) | 0.44 | 1.02 (0.90, 1.15) | | | 0.80 | |
| ***APOE ε4**Hanley-Cook** | 1.03 (0.85, 1.25) | 0.74 | 1.02 (0.84, 1.24) | | | 0.82 | |
| *APOE ε4* carriers | 1.01 (0.86, 1.17) | 0.95 | 1.03 (0.88, 1.20) | | | 0.73 | |
| *APOE ε4* non-carriers | 0.97 (0.86, 1.10) | 0.66 | 1.00 (0.89, 1.14) | | | 0.94 | |
| ***APOE ε4**Kesse-Guyot** | 1.04 (0.80, 1.34) | 0.77 | 1.02 (0.79, 1.33) | | | 0.86 | |
| *APOE ε4* carriers | 0.88 (0.72, 1.08) | 0.23 | 0.97 (0.79, 1.20) | | | 0.80 | |
| *APOE ε4* non-carriers | 0.85 (0.72, 1.01) | 0.059 | 0.95 (0.80, 1.13) | | | 0.58 | |
| ***APOE ε4**Stubbendorff** | 1.01 (0.78, 1.31) | 0.93 | 0.97 (0.75, 1.26) | | | 0.84 | |
| *APOE ε4* carriers | 0.88 (0.72, 1.09) | 0.24 | 0.94 (0.77, 1.16) | | | 0.57 | |
| *APOE ε4* non-carriers | 0.87 (0.74, 1.03) | 0.11 | 0.97 (0.82, 1.15) | | | 0.71 | |
| ***APOE ε4**Colizzi** | 1.14 (0.91, 1.43) | 0.24 | 1.12 (0.90, 1.40) | | | 0.31 | |
| *APOE ε4* carriers | 1.01 (0.85, 1.21) | 0.88 | 1.07 (0.89, 1.28) | | | 0.47 | |
| *APOE ε4* non-carriers | 0.85 (0.74, 0.98) | 0.027 | 0.95 (0.82, 1.10) | | | 0.49 | |
| ***APOE ε4**Bui** | 1.08 (0.81, 1.45) | 0.60 | 1.04 (0.78, 1.40) | | | 0.78 | |
| *APOE ε4* carriers | 0.87 (0.69, 1.10) | 0.24 | 0.96 (0.76, 1.22) | | | 0.76 | |
| *APOE ε4* non-carriers | 0.80 (0.67, 0.97) | 0.024 | 0.92 (0.76, 1.12) | | | 0.43 | |

Note: Interactions between seven EAT-Lancet diet scores and *APOE ε4* (yes/no) in relation to vascular dementia were investigated with Cox proportional hazard models. Model 1 was adjusted for age, sex, season, diet method version, and energy intake. Model 2 was adjusted for age, sex, season, diet method version, energy intake, education, smoking, alcohol consumption, physical activity, and BMI. Results are presented per 10% in increment scores. The scores were labelled based on first authors of the articles that reported the construction of the scores.

^a^There were 24,987 individuals with data on *APOE* ε4 status (17,444 non-carriers, 7,543 carriers). There were missing data on BMI (n = 37), and physical activity (n = 114). Out of the 409 with vascular dementia, 159 were *APOE ε4* carriers and 250 were *APOE ε4* non-carriers.

**^b^**Associations between the EAT-Lancet diet and vascular dementia is reported by *APOE ε4* status (carriers/non-carriers) based on results from the interaction analyses for each of the EAT-Lancet diet scores.

**Supplementary table 8. Associations between the EAT-Lancet diet and Aβ42 pathology.**

|  | **Aβ42 pathology n = 492** |  | | |  |  |
| --- | --- | --- | --- | --- | --- | --- |
| **N = 669** | **Model 1** | |  | **Model 2** | |  |
|  | **OR (95% CI)** | | **P-value** | **OR (95% CI)** | | **P-value** |
| **Knuppel** | 0.96 (0.77, 1.19) | | 0.70 | 0.94 (0.75, 1.17) | | 0.56 |
| **Trijsburg** | 1.00 (0.84, 1.19) | | 0.99 | 1.00 (0.84, 1.20) | | 0.98 |
| **Hanley-Cook** | 0.98 (0.83, 1.17) | | 0.84 | 0.97 (0.81, 1.15) | | 0.70 |
| **Kesse-Guyot** | 0.94 (0.75, 1.19) | | 0.62 | 0.90 (0.71, 1.16) | | 0.42 |
| **Stubbendorff** | 1.05 (0.81, 1.35) | | 0.72 | 1.03 (0.80, 1.34) | | 0.81 |
| **Colizzi** | 0.89 (0.73, 1.09) | | 0.26 | 0.87 (0.71, 1.08) | | 0.20 |
| **Bui** | 0.92 (0.70, 1.23) | | 0.58 | 0.87 (0.64, 1.17) | | 0.35 |

Note: Associations between seven EAT-Lancet diet scores and cerebrospinal fluid biomarker (CSF) Amyloid-β42 (Aβ42) was investigated with binary logistic regression analyses. Model 1 was adjusted for age, sex, season, diet method version, and energy intake. Model 2 was adjusted for age, sex, season, diet method version, energy intake, education, smoking, alcohol consumption, physical activity, and BMI. Results are presented per 10% in increment scores. The scores were labelled based on first authors of the articles that reported the construction of the scores. Three participants had missing data in model 2.

Because of a slight, assay-dependent drift in levels of CSF Amyloid-β42 (Aβ42) during the collection period 1995–2015, 2 different cutoffs for pathology were established for the period 1995–2003 (Aβ42 < 484.8 pg/mL) and 2004–2015 (Aβ42 < 577.1 pg/mL).

**Supplementary table 9. Associations between the EAT-Lancet diet and incident dementia without including BMI as a potential confounder.**

|  |  | | |  |  |  |  | |  | |  |  |
| --- | --- | --- | --- | --- | --- | --- | --- | --- | --- | --- | --- | --- |
|  | **Dementia (2014)**  **n = 1,783 (1,773)^a^** |  | **Dementia (2020)**  **n = 2,976 (2,963)^a^** | | |  | **Alzheimer’s disease**  **n = 1040 (1,034)^a^** |  | | **Vascular dementia**  **n = 426 (423)^a^** | |  |
| **N = 25,898** | **HR**  **(95% CI)** | **P-value** | **HR**  **(95% CI)** | | | **P-value** | **HR**  **(95% CI)** | **P-value** | | **HR**  **(95% CI)** | | **P-value** |
| **Knuppel** | 0.95  (0.89, 1.00) | 0.065 | 0.96  (0.92, 1.00) | | | 0.078 | 0.96  (0.89, 1.03) | 0.27 | | 0.97  (0.86, 1.09) | | 0.56 |
| **Trijsburg** | 0.97  (0.93, 1.02) | 0.19 | 0.99  (0.95, 1.02) | | | 0.52 | 0.98  (0.92, 1.04) | 0.50 | | 0.99  (0.90, 1.09) | | 0.83 |
| **Hanley-Cook** | 0.96  (0.91, 1.00) | 0.065 | 0.97  (0.94, 1.01) | | | 0.088 | 0.97  (0.91, 1.03) | 0.28 | | 1.01  (0.92, 1.11) | | 0.84 |
| **Kesse-Guyot** | 0.92  (0.87, 0.99) | 0.018 | 0.94  (0.90, 0.99) | | | 0.021 | 0.88  (0.80, 0.95) | 0.0021 | | 0.96  (0.84, 1.10) | | 0.54 |
| **Stubbendorff** | 0.97  (0.90, 1.03) | 0.29 | 0.99  (0.94, 1.04) | | | 0.59 | 0.98  (0.90, 1.07) | 0.69 | | 0.95  (0.83, 1.09) | | 0.51 |
| **Colizzi** | 0.97  (0.92, 1.02) | 0.22 | 0.97  (0.93, 1.02) | | | 0.23 | 0.96  (0.89, 1.03) | 0.26 | | 0.99  (0.88, 1.11) | | 0.86 |
| **Bui** | 0.93  (0.86, 1.01) | 0.077 | 0.97  (0.92, 1.03) | | | 0.37 | 0.94  (0.85, 1.04) | 0.20 | | 0.94  (0.80, 1.09) | | 0.41 |

Note: Associations between seven EAT-Lancet diet scores and all-cause dementia (yes/no) by 2014 (validated register data), and 2020 (validated register diagnoses until 2014, and unvalidated diagnoses between 2015-2020), Alzheimer’s disease, and vascular dementia were investigated with Cox proportional hazard models. The models were adjusted for age, sex, season, dietary method version, energy intake, education, smoking, alcohol consumption, and physical activity. Results are presented per 10% in increment scores. The scores were labelled based on first authors of the articles that reported the construction of the scores.

^a^There were missing data on smoking (n = 8), and physical activity (n = 120). Out of the participants with missing data, there were 10 participants with a dementia diagnosis in 2014, and 13 participants in 2020.

**Supplementary table 10. Associations between the EAT-Lancet diet and all-cause dementia when participants with diabetes were excluded.**

|  | **Dementia (2014)**  **n = 1,412 (1,409)^a^** | | |  |  |  | **Dementia (2020)**  **n = 2,357 (2,352)^a^** | |  | |  |  |
| --- | --- | --- | --- | --- | --- | --- | --- | --- | --- | --- | --- | --- |
| **N = 21,075** | **Model 1** |  | **Model 2** | | |  | **Model 1** |  | | **Model 2** | |  |
|  | **HR**  **(95% CI)** | **P-value** | **HR**  **(95% CI)** | | | **P-value** | **HR**  **(95% CI)** | **P-value** | | **HR**  **(95% CI)** | | **P-value** |
| **Knuppel** | 0.95  (0.89, 1.01) | 0.093 | 0.96  (0.90, 1.03) | | | 0.27 | 0.95  (0.90, 1.00) | 0.040 | | 0.97  (0.93, 1.01) | | 0.16 |
| **Trijsburg** | 0.97  (0.92, 1.02) | 0.17 | 0.98  (0.93, 1.03) | | | 0.40 | 0.98  (0.94, 1.02) | 0.33 | | 0.99  (0.96, 1.03) | | 0.74 |
| **Hanley-Cook** | 0.96  (0.91, 1.01) | 0.12 | 0.97  (0.92, 1.02) | | | 0.25 | 0.96  (0.93, 1.00) | 0.070 | | 0.97  (0.93, 1.01) | | 0.17 |
| **Kesse-Guyot** | 0.91  (0.85, 0.98) | 0.011 | 0.95  (0.88, 1.02) | | | 0.14 | 0.93  (0.88, 0.98) | 0.010 | | 0.96  (0.91, 1.02) | | 0.15 |
| **Stubbendorff** | 0.94  (0.87, 1.01) | 0.083 | 0.96  (0.89, 1.04) | | | 0.30 | 0.96  (0.91, 1.02) | 0.18 | | 0.99  (0.93, 1.04) | | 0.66 |
| **Colizzi** | 0.97  (0.91, 1.03) | 0.28 | 0.98  (0.92, 1.04) | | | 0.55 | 0.97  (0.92, 1.01) | 0.16 | | 0.98  (0.94, 1.03) | | 0.42 |
| **Bui** | 0.89  (0.82, 0.97) | 0.007 | 0.93  (0.85, 1.01) | | | 0.073 | 0.94  (0.88, 1.00) | 0.048 | | 0.97  (0.91, 1.04) | | 0.41 |

Note: Associations between seven EAT-Lancet diet scores and all-cause dementia (yes/no) by 2014 (validated register data), and 2020 (validated register diagnoses until 2014, and unvalidated diagnoses between 2015-2020) were investigated with Cox proportional hazard models. Model 1 was adjusted for age, sex, season, diet method version, and energy intake. Model 2 was adjusted for age, sex, season, dietary method version, energy intake, education, smoking, alcohol consumption, physical activity, and BMI. Results are presented per 10% in increment scores. The scores were labelled based on first authors of the articles that reported the construction of the scores. Participants with prevalent or incident diabetes during the study period were excluded.

^a^There were missing data on smoking (n = 7), BMI (n = 26), and physical activity (n = 87). Out of the participants with missing data, there were 3 participants with a dementia diagnosis in 2014, and 5 participants in 2020.

**Supplementary table 11. Associations of the EAT-Lancet diet with Alzheimer’s disease and vascular dementia when participants with diabetes were excluded.**

|  | **Alzheimer’s disease**  **n = 855 (853)^a^** | | |  |  |  | **Vascular dementia**  **n = 298 (297)^a^** | | |  |  |  |
| --- | --- | --- | --- | --- | --- | --- | --- | --- | --- | --- | --- | --- |
| **N = 21,075** | **Model 1** |  | **Model 2** |  | | | **Model 1** |  | **Model 2** | | |  |
|  | **HR**  **(95% CI)** | **P-value** | **HR**  **(95% CI)** | **P-value** | | | **HR**  **(95% CI)** | **P-value** | **HR**  **(95% CI)** | | | **P-value** |
| **Knuppel** | 0.97  (0.90, 1.06) | 0.54 | 0.98  (0.90, 1.06) | 0.57 | | | 0.91  (0.79, 1.05) | 0.20 | 1.00  (0.84, 1.12) | | | 0.66 |
| **Trijsburg** | 0.99  (0.92, 1.05) | 0.64 | 0.98  (0.92, 1.05) | 0.62 | | | 0.96  (0.86, 1.07) | 0.50 | 1.02  (0.91, 1.14) | | | 0.77 |
| **Hanley-Cook** | 0.99  (0.92, 1.05) | 0.65 | 0.99  (0.92, 1.06) | 0.67 | | | 0.95  (0.85, 1.07) | 0.42 | 0.99  (0.88, 1.11) | | | 0.88 |
| **Kesse-Guyot** | 0.88  (0.81, 0.97) | 0.0064 | 0.88  (0.80, 0.97) | 0.0082 | | | 0.92  (0.79, 1.08) | 0.31 | 1.04  (0.89, 1.22) | | | 0.65 |
| **Stubbendorff** | 0.99  (0.90, 1.08) | 0.77 | 0.99  (0.90, 1.08) | 0.77 | | | 0.86  (0.74, 1.01) | 0.068 | 0.94  (0.80, 1.10) | | | 0.44 |
| **Colizzi** | 1.00  (0.92, 1.08) | 0.95 | 0.99  (0.92, 1.07) | 0.84 | | | 0.92  (0.80, 1.05) | 0.20 | 0.98  (0.86, 1.12) | | | 0.77 |
| **Bui** | 0.92  (0.83, 1.03) | 0.14 | 0.92  (0.82, 1.03) | 0.13 | | | 0.83  (0.69, 0.99) | 0.038 | 0.94  (0.78, 1.13) | | | 0.52 |

Note: Associations between seven EAT-Lancet diet scores and Alzheimer’s disease (pure, and with vascular pathology), and vascular dementia were investigated with Cox proportional hazard models. Model 1 was adjusted for age, sex, season, diet method version, and energy intake. Model 2 was adjusted for age, sex, season, dietary method version, energy intake, education, smoking, alcohol consumption, physical activity, and BMI. Results are presented per 10% in increment scores. The scores were labelled based on first authors of the articles that reported the construction of the scores. Participants with prevalent or incident diabetes during the study period were excluded.

^a^There were missing data on smoking (n = 7), BMI (n = 26), and physical activity (n = 87). Out of the participants with missing data, there were 2 participants with Alzheimer’s disease, and 1 participant with vascular dementia.

**Supplementary table 12. Associations between the EAT-Lancet diet and all-cause dementia by *APOE* ε4 status when participants with diabetes were excluded.**

|  | **All-cause dementia n= 1,366 (1,363)**^a^ | | |  |  |  | |
| --- | --- | --- | --- | --- | --- | --- | --- |
| **N = 20,348** | **Model 1** |  | **Model 2** | | | |  |
| ***APOE ε4**EAT-Lancet diet scores^b^** | HR (95% CI) | P-value | HR (95% CI) | | | P-value | |
| ***APOE ε4**Knuppel** | 1.11 (0.99, 1.24) | 0.084 | 1.11 (0.98, 1.24) | | | 0.086 | |
| *APOE ε4* carriers | 0.99 (0.91, 1.08) | 0.80 | 1.00 (0.92, 1.09) | | | 0.92 | |
| *APOE ε4* non-carriers | 0.89 (0.82, 0.98) | 0.014 | 0.91 (0.83, 1.0) | | | 0.039 | |
| ***APOE ε4**Trijsburg** | 1.04 (0.94, 1.16) | 0.40 | 1.04 (0.94, 1.15) | | | 0.45 | |
| *APOE ε4* carriers | 0.97 (0.91, 1.04) | 0.47 | 0.98 (0.92, 1.06) | | | 0.66 | |
| *APOE ε4* non-carriers | 0.93 (0.87, 1.01) | 0.069 | 0.95 (0.88, 1.02) | | | 0.15 | |
| ***APOE ε4**Hanley-Cook** | 1.13 (1.02, 1.26) | 0.020 | 1.13 (1.02, 1.26) | | | 0.019 | |
| *APOE ε4* carriers | 1.03 (0.96, 1.10) | 0.46 | 1.04 (0.97, 1.12) | | | 0.31 | |
| *APOE ε4* non-carriers | 0.91 (0.84, 0.98) | 0.014 | 0.92 (0.85, 0.99) | | | 0.027 | |
| ***APOE ε4**Kesse-Guyot** | 1.07 (0.93, 1.22) | 0.36 | 1.06 (0.93, 1.22) | | | 0.39 | |
| *APOE ε4* carriers | 0.94 (0.85, 1.03) | 0.17 | 0.97 (0.88, 1.06) | | | 0.48 | |
| *APOE ε4* non-carriers | 0.88 (0.79, 0.97) | 0.013 | 0.91 (0.82, 1.01) | | | 0.078 | |
| ***APOE ε4**Stubbendorff** | 1.11 (0.96, 1.27) | 0.16 | 1.10 (0.96, 1.26) | | | 0.17 | |
| *APOE ε4* carriers | 0.97 (0.88, 1.07) | 0.59 | 0.99 (0.90, 1.10) | | | 0.89 | |
| *APOE ε4* non-carriers | 0.88 (0.79, 0.97) | 0.017 | 0.90 (0.81, 1.00) | | | 0.057 | |
| ***APOE ε4**Colizzi** | 1.11 (0.99, 1.25) | 0.083 | 1.11 (0.99, 1.25) | | | 0.077 | |
| *APOE ε4* carriers | 1.01 (0.93, 1.09) | 0.84 | 1.02 (0.94, 1.11) | | | 0.58 | |
| *APOE ε4* non-carriers | 0.91 (0.83, 0.99) | 0.033 | 0.92 (0.94, 1.00) | | | 0.066 | |
| ***APOE ε4**Bui** | 1.12 (0.96, 1.31) | 0.16 | 1.12 (0.96, 1.31) | | | 0.16 | |
| *APOE ε4* carriers | 0.93 (0.84, 1.04) | 0.22 | 0.96 (0.86, 1.08) | | | 0.53 | |
| *APOE ε4* non-carriers | 0.83 (0.74, 0.94) | 0.0027 | 0.86 (0.76, 0.97) | | | 0.016 | |

Note: Interactions between seven EAT-Lancet diet scores and *APOE ε4* (yes/no) in relation to all-cause dementia by 2014 were investigated with Cox proportional hazard models. Model 1 was adjusted for age, sex, season, diet method version, and energy intake. Model 2 was adjusted for age, sex, season, dietary method version, energy intake, education, smoking, alcohol consumption, physical activity, and BMI. Results are presented per 10% in increment scores. The scores were labelled based on first authors of the articles that reported the construction of the scores. Participants with prevalent or incident diabetes during the study period were excluded.

^a^There were missing data on smoking (n = 7), BMI (n = 23), and physical activity (n = 84). Out of the participants with missing data, there were 3 participants with a dementia diagnosis in 2014, and 3 participants in 2020.

**^b^**Associations between the EAT-Lancet diet and all-cause dementia is reported by *APOE ε4* status (carriers/non-carriers) based on results from the interaction analyses for each of the EAT-Lancet diet scores.

**Supplementary table 13.** **Associations between the EAT-Lancet diet and Alzheimer’s disease by *APOE* ε4 status when participants with diabetes were excluded.**

|  | **Alzheimer’s disease n = 833 (831)**^a^ | | |  |  |  |  |
| --- | --- | --- | --- | --- | --- | --- | --- |
| **N = 20,348^a^** | **Model 1** |  | **Model 2** | | |  | |
| ***APOE ε4**EAT-Lancet diet scores^b^** | HR (95% CI) | P-value | HR (95% CI) | | | P-value | |
| ***APOE ε4**Knuppel** | 1.11 (0.95, 1.29) | 0.18 | 1.12 (0.96, 1.30) | | | 0.15 | |
| *APOE ε4* carriers | 1.01 (0.91, 1.12) | 0.87 | 1.01 (0.91, 1.12) | | | 0.83 | |
| *APOE ε4* non-carriers | 0.91 (0.80, 1.03) | 0.14 | 0.90 (0.79, 1.03) | | | 0.13 | |
| ***APOE ε4**Trijsburg** | 1.07 (0.93, 1.22) | 0.35 | 1.07 (0.93, 1.22) | | | 0.36 | |
| *APOE ε4* carriers | 1.00 (0.92, 1.08) | 0.96 | 1.00 (0.92, 1.08) | | | 0.92 | |
| *APOE ε4* non-carriers | 0.94 (0.84, 1.04) | 0.23 | 0.94 (0.84, 1.04) | | | 0.64 | |
| ***APOE ε4**Hanley-Cook** | 1.15 (0.99, 1.32) | 0.051 | 1.16 (1.01, 1.33) | | | 0.037 | |
| *APOE ε4* carriers | 1.04 (0.96, 1.14) | 0.28 | 1.05 (0.97, 1.15) | | | 0.24 | |
| *APOE ε4* non-carriers | 0.91 (0.83, 1.02) | 0.11 | 0.91 (0.81, 1.02) | | | 0.092 | |
| ***APOE ε4**Kesse-Guyot** | 1.11 (0.92, 1.33) | 0.28 | 1.11 (0.92, 1.33) | | | 0.28 | |
| *APOE ε4* carriers | 0.91 (0.81, 1.02) | 0.092 | 0.91 (0.81, 1.02) | | | 0.097 | |
| *APOE ε4* non-carriers | 0.82 (0.71, 0.95) | 0.0083 | 0.82 (0.71, 0.95) | | | 0.0095 | |
| ***APOE ε4**Stubbendorff** | 1.11 (0.92, 1.33) | 0.27 | 1.12 (0.93, 1.34) | | | 0.24 | |
| *APOE ε4* carriers | 1.01 (0.90, 1.14) | 0.81 | 1.02 (0.90, 1.14) | | | 0.79 | |
| *APOE ε4* non-carriers | 0.92 (0.79, 1.06) | 0.24 | 0.91 (0.78, 1.06) | | | 0.22 | |
| ***APOE ε4**Colizzi** | 1.08 (0.92, 1.26) | 0.36 | 1.09 (0.93, 1.28) | | | 0.29 | |
| *APOE ε4* carriers | 1.01 (0.92, 1.12) | 0.79 | 1.01 (0.92, 1.12) | | | 0.81 | |
| *APOE ε4* non-carriers | 0.94 (0.83, 1.07) | 0.35 | 0.93 (0.82, 1.06) | | | 0.26 | |
| ***APOE ε4**Bui** | 1.09 (0.89, 1.35) | 0.39 | 1.11 (0.90, 1.37) | | | 0.32 | |
| *APOE ε4* carriers | 0.95 (0.83, 1.08) | 0.42 | 0.95 (0.83, 1.09) | | | 0.44 | |
| *APOE ε4* non-carriers | 0.86 (0.73, 1.02) | 0.092 | 0.85 (0.72, 1.01) | | | 0.071 | |

Note: Interactions between seven EAT-Lancet diet scores and *APOE ε4* (yes/no) in relation to Alzheimer’s disease were investigated with Cox proportional hazard models. Model 1 was adjusted for age, sex, season, diet method version, and energy intake. Model 2 was adjusted for age, sex, season, diet method version, energy intake, education, smoking, alcohol consumption, physical activity, and BMI. Results are presented per 10% in increment scores. The scores were labelled based on first authors of the articles that reported the construction of the scores. Participants with prevalent or incident diabetes during the study period were excluded.

^a^There were missing data on smoking (n = 7), BMI (n = 23), and physical activity (n = 84). Out of the participants with missing data, there were 2 participants with Alzheimer’s disease.

**^b^**Associations between the EAT-Lancet diet and Alzheimer’s disease is reported by *APOE ε4* status (carriers/non-carriers) based on results from the interaction analyses for each of the EAT-Lancet diet scores.

**Supplementary table 14. Associations between the EAT-Lancet diet and vascular dementia by *APOE* ε4 status when participants with diabetes were excluded.**

|  | **Vascular dementia n = 285 (284)**^a^ | | |  |  |  |  |
| --- | --- | --- | --- | --- | --- | --- | --- |
| **N = 20,348^a^** | **Model 1** |  | **Model 2** | | |  | |
| ***APOE ε4**EAT-Lancet diet scores^b^** | HR (95% CI) | P-value | HR (95% CI) | | | P-value | |
| ***APOE ε4**Knuppel** | 1.00 (0.77, 1.28) | 0.97 | 0.97 (0.75, 1.25) | | | 0.83 | |
| *APOE ε4* carriers | 0.92 (0.75, 1.13) | 0.43 | 0.96 (0.78, 1.18) | | | 0.68 | |
| *APOE ε4* non-carriers | 0.93 (0.77, 1.11) | 0.40 | 0.98 (0.82, 1.18) | | | 0.86 | |
| ***APOE ε4**Trijsburg** | 0.97 (0.77, 1.21) | 0.76 | 0.94 (0.76, 1.18) | | | 0.63 | |
| *APOE ε4* carriers | 0.93 (0.79, 1.11) | 0.43 | 0.97 (0.82, 1.15) | | | 0.73 | |
| *APOE ε4* non-carriers | 0.97 (0.84, 1.12) | 0.65 | 1.03 (0.89, 1.19) | | | 0.74 | |
| ***APOE ε4**Hanley-Cook** | 1.07 (0.85, 1.35) | 0.55 | 1.06 (0.84, 1.34) | | | 0.60 | |
| *APOE ε4* carriers | 1.01 (0.84, 1.20) | 0.93 | 1.04 (0.87, 1.24) | | | 0.69 | |
| *APOE ε4* non-carriers | 0.94 (0.81, 1.09) | 0.42 | 0.98 (0.84, 1.14) | | | 0.74 | |
| ***APOE ε4**Kesse-Guyot** | 0.96 (0.71, 1.31) | 0.80 | 0.94 (0.70, 1.28) | | | 0.70 | |
| *APOE ε4* carriers | 0.92 (0.72, 1.15) | 0.44 | 1.01 (0.79, 1.28) | | | 0.96 | |
| *APOE ε4* non-carriers | 0.95 (0.77, 1.16) | 0.61 | 1.07 (0.87, 1.31) | | | 0.53 | |
| ***APOE ε4**Stubbendorff** | 0.97 (0.71, 1.32) | 0.84 | 0.93 (0.68, 1.26) | | | 0.63 | |
| *APOE ε4* carriers | 0.85 (0.67, 1.08) | 0.18 | 0.89 (0.70, 1.14) | | | 0.37 | |
| *APOE ε4* non-carriers | 0.88 (0.71, 1.08) | 0.21 | 0.96 (0.78, 1.19) | | | 0.74 | |
| ***APOE ε4**Colizzi** | 1.10 (0.85, 1.45) | 0.47 | 1.09 (0.83, 1.42) | | | 0.54 | |
| *APOE ε4* carriers | 0.97 (0.79, 1.19) | 0.80 | 1.03 (0.84, 1.27) | | | 0.78 | |
| *APOE ε4* non-carriers | 0.88 (0.74, 1.05) | 0.16 | 0.95 (0.79, 1.13) | | | 0.56 | |
| ***APOE ε4**Bui** | 1.04 (0.73, 1.47) | 0.84 | 0.99 (0.70, 1.41) | | | 0.98 | |
| *APOE ε4* carriers | 0.85 (0.65, 1.12) | 0.24 | 0.94 (0.71, 1.23) | | | 0.63 | |
| *APOE ε4* non-carriers | 0.82 (0.65, 1.04) | 0.10 | 0.94 (0.74, 1.20) | | | 0.62 | |

Note: Interactions between seven EAT-Lancet diet scores and *APOE ε4* (yes/no) in relation to vascular dementia were investigated with Cox proportional hazard models. Model 1 was adjusted for age, sex, season, diet method version, and energy intake. Model 2 was adjusted for age, sex, season, diet method version, energy intake, education, smoking, alcohol consumption, physical activity, and BMI. Results are presented per 10% in increment scores. The scores were labelled based on first authors of the articles that reported the construction of the scores. Participants with prevalent or incident diabetes during the study period were excluded.

^a^There were missing data on smoking (n = 7), BMI (n = 23), and physical activity (n = 84). Out of the participants with missing data, there was 1 participant with vascular dementia.

**^b^**Associations between the EAT-Lancet diet and vascular dementia is reported by *APOE ε4* status (carriers/non-carriers) based on results from the interaction analyses for each of the EAT-Lancet diet scores.

**Supplementary table 15. Associations between the EAT-Lancet diet and all-cause dementia when participants with incident dementia <5 years from baseline were excluded.**

|  | **Dementia (2014)**  **n = 1,716 (1,706)^a^** | | |  |  |  | **Dementia (2020)**  **n = 2,909 (2,896)^a^** | |  | |  |  |
| --- | --- | --- | --- | --- | --- | --- | --- | --- | --- | --- | --- | --- |
| **N = 25,831** | **Model 1** |  | **Model 2** | | |  | **Model 1** |  | | **Model 2** | |  |
|  | **HR**  **(95% CI)** | **P-value** | **HR**  **(95% CI)** | | | **P-value** | **HR**  **(95% CI)** | **P-value** | | **HR**  **(95% CI)** | | **P-value** |
| **Knuppel** | 0.94  (0.89, 0.99) | 0.034 | 0.96  (0.90, 1.02) | | | 0.16 | 0.95  (0.91, 0.99) | 0.027 | | 0.97  (0.93, 1.01) | | 0.16 |
| **Trijsburg** | 0.97  (0.92, 1.01) | 0.13 | 0.98  (0.94, 1.03) | | | 0.41 | 0.98  (0.95, 1.02) | 0.28 | | 0.99  (0.96, 1.03) | | 0.80 |
| **Hanley-Cook** | 0.95  (0.91, 1.00) | 0.051 | 0.96  (0.92, 1.01) | | | 0.13 | 0.96  (0.93, 0.99) | 0.045 | | 0.97  (0.94, 1.01) | | 0.14 |
| **Kesse-Guyot** | 0.90  (0.85, 0.96) | 0.0016 | 0.94  (0.88, 1.00) | | | 0.061 | 0.92  (0.88, 0.97) | 0.0011 | | 0.95  (0.91, 1.00) | | 0.060 |
| **Stubbendorff** | 0.95  (0.89, 1.02) | 0.15 | 0.98  (0.92, 1.05) | | | 0.61 | 0.97  (0.92, 1.02) | 0.22 | | 0.99  (0.95, 1.05) | | 0.94 |
| **Colizzi** | 0.96  (0.91, 1.01) | 0.13 | 0.98  (0.92, 1.03) | | | 0.40 | 0.96  (0.92, 1.01) | 0.085 | | 0.98  (0.94, 1.02) | | 0.37 |
| **Bui** | 0.92  (0.85, 0.99) | 0.029 | 0.96  (0.89, 1.04) | | | 0.32 | 0.95  (0.90, 1.01) | 0.090 | | 0.99  (0.94, 1.05) | | 0.79 |

Associations between seven EAT-Lancet diet scores and all-cause dementia (yes/no) by 2014 (validated register data), and 2020 (validated register diagnoses until 2014, and unvalidated diagnoses between 2015-2020) were investigated with Cox proportional hazard models. Model 1 was adjusted for age, sex, season, diet method version, and energy intake. Model 2 was adjusted for age, sex, season, dietary method version, energy intake, education, smoking, alcohol consumption, physical activity, and BMI. Results are presented per 10% in increment scores. The scores were labelled based on first authors of the articles that reported the construction of the scores.

^a^There were missing data on smoking (n = 8), BMI (n = 40), and physical activity (n = 120). Out of the participants with missing data, there were 10 participants with a dementia diagnosis in 2014, and 13 participants in 2020.

**Supplementary table 16. Associations of the EAT-Lancet diet with Alzheimer’s disease and vascular dementia when participants with incident dementia <5 years from baseline were excluded.**

|  | **Alzheimer’s disease**  **n = 1003 (997)^a^** | | |  |  |  | **Vascular dementia**  **n = 408 (405)^a^** | | |  |  |  |
| --- | --- | --- | --- | --- | --- | --- | --- | --- | --- | --- | --- | --- |
| **N = 25,831** | **Model 1** |  | **Model 2** |  | | | **Model 1** |  | **Model 2** | | |  |
|  | **HR**  **(95% CI)** | **P-value** | **HR**  **(95% CI)** | **P-value** | | | **HR**  **(95% CI)** | **P-value** | **HR**  **(95% CI)** | | | **P-value** |
| **Knuppel** | 0.96  (0.89, 1.04) | 0.31 | 0.96  (0.89, 1.04) | 0.32 | | | 0.93  (0.82, 1.04) | 0.20 | 0.98  (0.87, 1.11) | | | 0.74 |
| **Trijsburg** | 0.99  (0.93, 1.05) | 0.74 | 0.99  (0.93, 1.05) | 0.72 | | | 0.94  (0.86, 1.03) | 0.21 | 1.00  (0.90, 1.10) | | | 0.92 |
| **Hanley-Cook** | 0.98  (0.92, 1.04) | 0.43 | 0.98  (0.92, 1.04) | 0.44 | | | 0.99  (0.90, 1.09) | 0.79 | 1.02  (0.92, 1.12) | | | 0.74 |
| **Kesse-Guyot** | 0.89  (0.82, 0.96) | 0.0040 | 0.88  (0.81, 0.96) | 0.0053 | | | 0.87  (0.76, 0.99) | 0.032 | 0.97  (0.84, 1.11) | | | 0.61 |
| **Stubbendorff** | 0.99  (0.92, 1.09) | 0.96 | 1.00  (0.91, 1.09) | 0.94 | | | 0.89  (0.77, 1.01) | 0.073 | 0.97  (0.84, 1.11) | | | 0.64 |
| **Colizzi** | 0.97  (0.91, 1.05) | 0.45 | 0.97  (0.90, 1.04) | 0.41 | | | 0.94  (0.84, 1.05) | 0.27 | 1.00  (0.90, 1.13) | | | 0.95 |
| **Bui** | 0.96  (0.87, 1.06) | 0.40 | 0.96  (0.86, 1.06) | 0.39 | | | 0.86  (0.74, 1.00) | 0.050 | 0.98  (0.84, 1.15) | | | 0.80 |

Associations between seven EAT-Lancet diet scores and Alzheimer’s disease (pure, and with vascular pathology), and vascular dementia were investigated with Cox proportional hazard models. Model 1 was adjusted for age, sex, season, diet method version, and energy intake. Model 2 was adjusted for age, sex, season, dietary method version, energy intake, education, smoking, alcohol consumption, physical activity, and BMI. Results are presented per 10% in increment scores. The scores were labelled based on first authors of the articles that reported the construction of the scores.

^a^There were missing data on smoking (n = 8), BMI (n = 40), and physical activity (n = 120). Out of the participants with missing data, there were 5 participants with Alzheimer’s disease, and 3 participants with vascular dementia.

**Supplementary table 17. Associations between the EAT-Lancet diet and all-cause dementia by *APOE* ε4 status when participants with incident dementia <5 years from baseline were excluded.**

|  | **All-cause dementia n= 1,663 (1,655)**^a^ | | |  |  |  | |
| --- | --- | --- | --- | --- | --- | --- | --- |
| **N = 24,922** | **Model 1** |  | **Model 2** | | | |  |
| ***APOE ε4**EAT-Lancet diet scores^b^** | HR (95% CI) | P-value | HR (95% CI) | | | P-value | |
| ***APOE ε4**Knuppel** | 1.11 (1.00, 1.23) | 0.057 | 1.11 (1.00, 1.23) | | | 0.057 | |
| *APOE ε4* carriers | 0.98 (0.91, 1.06) | 0.70 | 1.00 (0.93, 1.08) | | | 0.95 | |
| *APOE ε4* non-carriers | 0.89 (0.82, 0.96) | 0.0040 | 0.91 (0.84, 0.98) | | | 0.016 | |
| ***APOE ε4**Trijsburg** | 1.02 (0.93, 1.12) | 0.67 | 1.02 (0.93, 1.11) | | | 0.75 | |
| *APOE ε4* carriers | 0.97 (0.91, 1.03) | 0.34 | 0.98 (0.92, 1.05) | | | 0.55 | |
| *APOE ε4* non-carriers | 0.95 (0.89, 1.01) | 0.12 | 0.97 (0.90, 1.03) | | | 0.31 | |
| ***APOE ε4**Hanley-Cook** | 1.11 (1.01, 1.22) | 0.033 | 1.11 (1.01, 1.22) | | | 0.027 | |
| *APOE ε4* carriers | 1.01 (0.95, 1.08) | 0.77 | 1.02 (0.96, 1.09) | | | 0.52 | |
| *APOE ε4* non-carriers | 0.91 (0.85, 0.98) | 0.0079 | 0.92 (0.86, 0.98) | | | 0.016 | |
| ***APOE ε4**Kesse-Guyot** | 1.11 (0.98, 1.26) | 0.11 | 1.11 (0.97, 1.24) | | | 0.15 | |
| *APOE ε4* carriers | 0.95 (0.87, 1.03) | 0.23 | 0.98 (0.90, 1.07) | | | 0.66 | |
| *APOE ε4* non-carriers | 0.86 (0.78, 0.94) | 0.00087 | 0.89 (0.81, 0.98) | | | 0.019 | |
| ***APOE ε4**Stubbendorff** | 1.09 (0.96, 1.24) | 0.17 | 1.08 (0.95, 1.23) | | | 0.22 | |
| *APOE ε4* carriers | 0.99 (0.90, 1.08) | 0.75 | 1.01 (0.92, 1.11) | | | 0.82 | |
| *APOE ε4* non-carriers | 0.90 (0.82, 0.99) | 0.031 | 0.93 (0.85, 1.03) | | | 0.15 | |
| ***APOE ε4**Colizzi** | 1.07 (0.96, 1.19) | 0.21 | 1.07 (0.96, 1.19) | | | 0.22 | |
| *APOE ε4* carriers | 0.99 (0.91, 1.06) | 0.70 | 1.00 (0.93, 1.08) | | | 0.98 | |
| *APOE ε4* non-carriers | 0.92 (0.85, 0.99) | 0.035 | 0.94 (0.86, 1.01) | | | 0.099 | |
| ***APOE ε4**Bui** | 1.12 (0.98, 1.29) | 0.11 | 1.12 (0.97, 1.29) | | | 0.13 | |
| *APOE ε4* carriers | 0.97 (0.87, 1.07) | 0.51 | 1.00 (0.90, 1.12) | | | 0.93 | |
| *APOE ε4* non-carriers | 0.86 (0.77, 0.96) | 0.0048 | 0.90 (0.81, 1.00) | | | 0.054 | |

Note: Interactions between seven EAT-Lancet diet scores and *APOE ε4* (yes/no) in relation to all-cause dementia by 2014 were investigated with Cox proportional hazard models. Model 1 was adjusted for age, sex, season, diet method version, and energy intake. Model 2 was adjusted for age, sex, season, dietary method version, energy intake, education, smoking, alcohol consumption, physical activity, and BMI. Results are presented per 10% in increment scores. The scores were labelled based on first authors of the articles that reported the construction of the scores.

^a^There were missing data on BMI (n = 37), and physical activity (n = 114). Out of the participants with missing data, there were 8 participants with a dementia diagnosis.

**^b^**Associations between the EAT-Lancet diet and all-cause dementia is reported by *APOE ε4* status (carriers/non-carriers) based on results from the interaction analyses for each of the EAT-Lancet diet scores.

**Supplementary table 18. Associations between the EAT-Lancet diet and Alzheimer’s disease by *APOE* ε4 status when participants with incident dementia <5 years from baseline were excluded.**

|  | **Alzheimer’s disease n = 976 (972)**^a^ | | |  |  |  |  |
| --- | --- | --- | --- | --- | --- | --- | --- |
| **N = 24,922^a^** | **Model 1** |  | **Model 2** | | |  | |
| ***APOE ε4**EAT-Lancet diet scores^b^** | HR (95% CI) | P-value | HR (95% CI) | | | P-value | |
| ***APOE ε4**Knuppel** | 1.10 (0.96, 1.27) | 0.17 | 1.11 (0.97, 1.28) | | | 0.13 | |
| *APOE ε4* carriers | 1.00 (0.91, 1.10) | 0.95 | 1.00 (0.91, 1.10) | | | 0.97 | |
| *APOE ε4* non-carriers | 0.90 (0.81, 1.01) | 0.082 | 0.90 (0.80, 1.00) | | | 0.060 | |
| ***APOE ε4**Trijsburg** | 1.02 (0.90, 1.15) | 0.78 | 1.02 (0.90, 1.15) | | | 0.75 | |
| *APOE ε4* carriers | 0.99 (0.92, 1.07) | 0.86 | 0.99 (0.92, 1.07) | | | 0.84 | |
| *APOE ε4* non-carriers | 0.98 (0.89, 1.07) | 0.61 | 0.97 (0.88, 1.07) | | | 0.56 | |
| ***APOE ε4**Hanley-Cook** | 1.13 (0.99, 1.28) | 0.065 | 1.14 (1.01, 1.30) | | | 0.037 | |
| *APOE ε4* carriers | 1.03 (0.95, 1.12) | 0.48 | 1.04 (0.96, 1.12) | | | 0.39 | |
| *APOE ε4* non-carriers | 0.92 (0.83, 1.01) | 0.076 | 0.91 (0.82, 1.00) | | | 0.052 | |
| ***APOE ε4**Kesse-Guyot** | 1.12 (0.95, 1.32) | 0.19 | 1.11 (0.94, 1.31) | | | 0.21 | |
| *APOE ε4* carriers | 0.93 (0.83, 1.03) | 0.15 | 0.92 (0.83, 1.03) | | | 0.14 | |
| *APOE ε4* non-carriers | 0.83 (0.73, 0.95) | 0.0051 | 0.83 (0.72, 0.95) | | | 0.0067 | |
| ***APOE ε4**Stubbendorff** | 1.07 (0.91, 1.27) | 0.40 | 1.08 (0.92, 1.28) | | | 0.35 | |
| *APOE ε4* carriers | 1.02 (0.91, 1.13) | 0.76 | 1.02 (0.91, 1.14) | | | 0.75 | |
| *APOE ε4* non-carriers | 0.95 (0.83, 1.08) | 0.42 | 0.94 (0.82, 1.08) | | | 0.37 | |
| ***APOE ε4**Colizzi** | 1.01 (0.88, 1.17) | 0.85 | 1.03 (0.89, 1.19) | | | 0.71 | |
| *APOE ε4* carriers | 0.97 (0.88, 1.06) | 0.52 | 0.97 (0.88, 1.06) | | | 0.52 | |
| *APOE ε4* non-carriers | 0.96 (0.86, 1.07) | 0.44 | 0.94 (0.84, 1.06) | | | 0.32 | |
| ***APOE ε4**Bui** | 1.08 (0.89, 1.31) | 0.43 | 1.09 (0.90, 1.32) | | | 0.36 | |
| *APOE ε4* carriers | 0.98 (0.86, 1.11) | 0.74 | 0.98 (0.86, 1.11) | | | 0.77 | |
| *APOE ε4* non-carriers | 0.91 (0.78, 1.06) | 0.21 | 0.90 (0.77, 1.01) | | | 0.17 | |

Note: Interactions between seven EAT-Lancet diet scores and *APOE ε4* (yes/no) in relation to Alzheimer’s disease were investigated with Cox proportional hazard models. Model 1 was adjusted for age, sex, season, diet method version, and energy intake. Model 2 was adjusted for age, sex, season, diet method version, energy intake, education, smoking, alcohol consumption, physical activity, and BMI. Results are presented per 10% in increment scores. The scores were labelled based on first authors of the articles that reported the construction of the scores.

^a^There were missing data on BMI (n = 37), and physical activity (n = 114). Out of the participants with missing data, there were 4 participants with Alzheimer’s disease

**^b^**Associations between the EAT-Lancet diet and Alzheimer’s disease is reported by *APOE ε4* status (carriers/non-carriers) based on results from the interaction analyses for each of the EAT-Lancet diet scores.

**Supplementary table 19. Associations between the EAT-Lancet diet and vascular dementia by *APOE* ε4 status when participants with incident dementia <5 years from baseline were excluded.**

|  | **Vascular dementia n = 394 (391)**^a^ | | |  |  |  |  |
| --- | --- | --- | --- | --- | --- | --- | --- |
| **N = 24,922^a^** | **Model 1** |  | **Model 2** | | |  | |
| ***APOE ε4**EAT-Lancet diet scores^b^** | HR (95% CI) | P-value | HR (95% CI) | | | P-value | |
| ***APOE ε4**Knuppel** | 1.02 (0.82, 1.27) | 0.83 | 1.00 (0.81, 1.25) | | | 0.97 | |
| *APOE ε4* carriers | 0.95 (0.79, 1.13 | 0.56 | 0.99 (0.82, 1.18) | | | 0.89 | |
| *APOE ε4* non-carriers | 0.93 (0.80, 1.07) | 0.30 | 0.98 (0.85, 1.14) | | | 0.82 | |
| ***APOE ε4**Trijsburg** | 0.96 (0.80, 1.17) | 0.70 | 0.94 (0.78, 1.14) | | | 0.54 | |
| *APOE ε4* carriers | 0.91 (0.79, 1.06) | 0.24 | 0.95 (0.82, 1.11) | | | 0.52 | |
| *APOE ε4* non-carriers | 0.95 (0.84, 1.07) | 0.40 | 1.01 (0.89, 1.14) | | | 0.88 | |
| ***APOE ε4**Hanley-Cook** | 1.05 (0.86, 1.28) | 0.64 | 1.04 (0.85, 1.26) | | | 0.71 | |
| *APOE ε4* carriers | 1.03 (0.88, 1.20) | 0.75 | 1.05 (0.90, 1.22) | | | 0.56 | |
| *APOE ε4* non-carriers | 0.98 (0.86, 1.11) | 0.74 | 1.01 (0.89, 1.14) | | | 0.89 | |
| ***APOE ε4**Kesse-Guyot** | 1.04 (0.80, 1.36) | 0.75 | 1.03 (0.79, 1.34) | | | 0.84 | |
| *APOE ε4* carriers | 0.89 (0.73, 1.10) | 0.29 | 0.98 (0.80, 1.21) | | | 0.87 | |
| *APOE ε4* non-carriers | 0.86 (0.72, 1.02) | 0.079 | 0.96 (0.80, 1.14) | | | 0.62 | |
| ***APOE ε4**Stubbendorff** | 1.02 (0.78, 1.33) | 0.89 | 0.98 (0.75, 1.28) | | | 0.88 | |
| *APOE ε4* carriers | 0.90 (0.73, 1.11) | 0.32 | 0.96 (0.77, 1.18) | | | 0.68 | |
| *APOE ε4* non-carriers | 0.88 (0.74, 1.05) | 0.16 | 0.98 (0.82, 1.16) | | | 0.79 | |
| ***APOE ε4**Colizzi** | 1.13 (0.90, 1.42) | 0.30 | 1.10 (0.88, 1.39) | | | 0.40 | |
| *APOE ε4* carriers | 1.01 (0.85, 1.21) | 0.88 | 1.07 (0.89, 1.28) | | | 0.47 | |
| *APOE ε4* non-carriers | 0.90 (0.78, 1.04) | 0.15 | 0.97 (0.84, 1.12) | | | 0.67 | |
| ***APOE ε4**Bui** | 1.06 (0.79, 1.43) | 0.70 | 1.02 (0.76, 1.38) | | | 0.89 | |
| *APOE ε4* carriers | 0.89 (0.71, 1.13) | 0.36 | 0.99 (0.78, 1.26) | | | 0.93 | |
| *APOE ε4* non-carriers | 0.84 (0.69, 1.03) | 0.088 | 0.97 (0.79, 1.19) | | | 0.76 | |

Note: Interactions between seven EAT-Lancet diet scores and *APOE ε4* (yes/no) in relation to vascular dementia were investigated with Cox proportional hazard models. Model 1 was adjusted for age, sex, season, diet method version, and energy intake. Model 2 was adjusted for age, sex, season, diet method version, energy intake, education, smoking, alcohol consumption, physical activity, and BMI. Results are presented per 10% in increment scores. The scores were labelled based on first authors of the articles that reported the construction of the scores.

^a^There were missing data on BMI (n = 37), and physical activity (n = 114). Out of the participants with missing data, there were 3 participants with vascular dementia.

**^b^**Associations between the EAT-Lancet diet and vascular dementia is reported by *APOE ε4* status (carriers/non-carriers) based on results from the interaction analyses for each of the EAT-Lancet diet scores.
